# A Multimodal Lifestyle Psychosocial Survivorship Program in Young Cancer Survivors: The CARE for CAYA Program—A Randomized Clinical Trial Embedded in a Longitudinal Cohort Study

**DOI:** 10.1001/jamanetworkopen.2024.2375

**Published:** 2024-03-25

**Authors:** Julia von Grundherr, Simon Elmers, Barbara Koch, Lesley-Ann Hail, Julia Mann, Gabriele Escherich, Corinna Bergelt, Luisa Samland, Wiebke Jensen, Eik Vettorazzi, Maria Stark, Luzia Valentini, Freerk T. Baumann, Susanne Singer, Rüdiger Reer, Ronja Beller, Gabriele Calaminus, Jörg Faber, Carl Friedrich Classen, Judith Gebauer, Inken Hilgendorf, Michael Koehler, Alexander Puzik, Nicole Salzmann, Annette Sander, Lisa Schiffmann, Magdalena Sokalska-Duhme, Sonja Schuster, Ann-Kristin Kock-Schoppenhauer, Carsten Bokemeyer, Marianne Sinn, Alexander Stein, Sarah Dwinger, Jannike Salchow

**Affiliations:** 1 Department of Oncology, Hematology, BMT with Section Pneumology, Hubertus Wald Tumor Center, University Cancer Center Hamburg, University Medical Center Hamburg-Eppendorf, Hamburg, Germany; 2 Department of Pediatric Hematology and Oncology, Hubertus Wald Tumor Center, University Cancer Center Hamburg, University Medical Center Hamburg-Eppendorf, Hamburg, Germany; 3 Department for Medical Psychology, University Medical Center Hamburg-Eppendorf, Hamburg, Germany; 4 Institute for Medical Psychology, Greifswald University Medicine, Greifswald, Germany; 5 Institute of Medical Biometry and Epidemiology, University Medical Center Hamburg-Eppendorf, Hamburg, Germany.; 6 Faculty of Agriculture and Food Sciences, University of Applied Sciences, Neubrandenburg, Germany; 7 Department I of Internal Medicine, Center for Integrated Oncology Aachen Bonn Cologne Dusseldorf, University Hospital of Cologne, Cologne, Germany; 8 Institute for Medical Biostatistics, Epidemiology and Informatics, Division of Epidemiology and Health Services Research, University Medical Centre, Johannes Gutenberg University, Mainz, Germany; 9 Institute of Human Movement Science, Faculty of Psychology and Human Movement, University of Hamburg, Hamburg, Germany; 10 Clinic for Pediatrics III, Department of Pediatric Hematology/Oncology, West German Cancer Centre, University Hospital Essen, Essen, Germany; 11 Department of Pediatric Hematology and Oncology, University Hospital Bonn, Venusberg Campus 1, Bonn, Germany; 12 Department of Pediatric Hematology/Oncology, Center for Pediatric and Adolescent Medicine, University Cancer Center, University Medical Center of the Johannes Gutenberg-University Mainz, Mainz, Germany; 13 Pediatric Oncology and Palliative Care Section, University Medicine Rostock, Childrens' and Adolescents Hospital Rostock, Rostock, Germany; 14 Department of Internal Medicine I, University Hospital of Schleswig-Holstein, Campus Lübeck, Lübeck, Germany; 15 Klinik für Innere Medizin II, Abteilung für Hämatologie und Internistische Onkologie, Universitätsklinikum Jena, Am Klinikum 1, Jena, Deutschland; 16 Department of Hematology and Oncology, University Hospital Magdeburg, Magdeburg, Germany; 17 Specialty Practice for Psycho-Oncology, Magdeburg, Germany; 18 Department of Pediatric Hematology and Oncology, Medical Center, University of Freiburg, Faculty of Medicine, University of Freiburg, Freiburg, Germany; 19 Pediatric Hematology and Oncology, University Children´s Hospital Muenster, Albert-Schweitzer Campus 1, Muenster, Germany; 20 Pediatric Hematology and Oncology, Hannover Medical School, Hannover, Germany; 21 Institute of Clinical Epidemiology and Biometry, Julius-Maximilians-Universität Würzburg, CCC WERA, University Hospital Würzburg, Würzburg, Germany; 22 Department of Pediatric Hematology, Oncology & Immunology, Olgahospital, Klinikum Stuttgart, Stuttgart, Germany; 23 Pediatric Hematology and Oncology, Department of Pediatrics, Friedrich-Alexander University of Erlangen-Nürnberg, Erlangen, Germany; 24 IT Center for Clinical Research, Universität zu Lübeck, Lübeck, Germany

## Abstract

**Importance:**

There is a lack of trials examining the effect of counseling interventions for child, adolescent, and younger adult (CAYA) cancer survivors.

**Objective:**

To assess lifestyle habits and the psychosocial situation of CAYAs to determine the efficacy of needs-based interventions in the CARE for CAYA program (CFC-P).

**Design, Setting, and Participants:**

The CFC-P was conducted as a multicenter program in 14 German outpatient clinics, mainly university cancer centers. Recruitment began January 1, 2018; a randomized clinical trial was conducted until July 15, 2019; and intervention was continued as a longitudinal cohort study until March 31, 2021. Data preparation was conducted from April 1, 2021, and analysis was conducted from August 14, 2021, to May 31, 2022. Herein, predefined confirmatory analyses pertain to the RCT and descriptive results relate to the overall longitudinal study. Data analysis was based on the full analysis set, which is as close as possible to the intention-to-treat principle.

**Intervention:**

A comprehensive assessment determined needs in physical activity, nutrition and psychooncology. Those with high needs participated in 1 to 3 modules. In the RCT, the IG received 5 counseling sessions plus newsletters, while the control group CG received 1 counseling session.

**Main Outcomes and Measures:**

The primary outcome was the change in the rate of CAYAs with high needs at 52 weeks. Secondary outcomes were feasibility, modular-specific end points, satisfaction, quality of life, and fatigue.

**Results:**

Of 1502 approached CAYAs aged 15 to 39 years, 692 declined participation. Another 22 CAYAs were excluded, resulting in 788 participants. In the randomized clinical trial, 359 CAYAs were randomized (intervention group [IG], n = 183; control group [CG], n = 176), and 274 were followed up. In the RCT, the median age was 25.0 (IQR, 19.9-32.2) years; 226 were female (63.0%) and 133 male (37.0%). After 52 weeks, 120 CAYAs (87.0%) in the IG and 115 (86.5%) in the CG still had a high need in at least 1 module (odds ratio, 1.04; 95% CI, 0.51-2.11; *P* = .91). Both groups reported reduced needs, improved quality of life, reduced fatigue, and high satisfaction with the CFC-P.

**Conclusions and Relevance:**

In this randomized clinical trial, the implementation of a lifestyle program in this cohort was deemed necessary, despite not meeting the primary outcome. The interventions did not alter the rate of high needs. The results may provide guidance for the development of multimodal interventions in the follow-up care of CAYAs.

**Trial Registration:**

German Clinical Trial Register: DRKS00012504

## Key Points

**Question:**

Can a multimodal survivorship lifestyle program with counseling interventions physical activity, nutrition, and psychooncology improve the health behaviors and psychosocial situation in children, adolescents, and younger adults (CAYAs) who are cancer survivors?

**Findings:**

This randomized clinical trial including 359 CAYAs with a high need for intervention did not show a significant difference between the cohorts who received intervention and the controls. However, both groups reported reduced needs, improved quality of life, reduced fatigue, and high satisfaction with the program.

**Meaning:**

The findings of this trial suggest that the needs of CAYAs are complex, and further studies are needed to better understand which specific interventions are effective for this group.

## Teaser

This randomized clinical trial followed by a longitudinal study evaluated the use of needs-based interventions including physical activity, nutrition, and psychoongology in children, adolescent, and younger adult cancer survivors.

## Introduction

Approximately half a million patients are diagnosed with cancer in Germany every year, and about 4% are between ages 0 and 39 years at the time of diagnosis,^1^ referred to as children, adolescents and younger adults (CAYAs).^2,3^ Survival rates for CAYAs have improved to more than 80%.^4^ However, adolescent and younger adult cancer survivors face an increased risk of long-term sequelae. They have physical and psychosocial problems,^5^ a higher risk of recurrence,^6^ secondary cancer,^7^ cardiovascular events,^8,9^ distress,^10^ psychological disorders,^11^ and a reduced quality of life (QOL).^12,13^ These sequelae persist long after acute cancer-related therapy cessation and are associated with unemployment.^14^ How to detect long-term subsequent central nervous system neoplasms^15^ and cardiovascular risks^16^ after careful consideration of the potential harms and benefits of surveillance for CAYAs is another important aspect for health care professionals.

Although a healthy lifestyle can reduce the risk for cardiovascular events^17,18^ and has a positive impact on health-related QOL,^19,20^ surveys of cancer survivors’ health behaviors show that a considerable proportion do not engage in sufficient physical activity^21,22,23^ and adhere to a balanced diet.^24,25^ Personalized physical activity or nutritional counseling, as well as age-appropriate interventions, are described to improve health behavior, QOL, and the psychosocial situation of CAYAs,^24,26,27,28,29^ but evidence is still sparse.

Cancer survivorship guidelines recommend comprehensive follow-up care by multidisciplinary teams.^2,30^ In Germany, consultations with integrated psychosocial care exist,^31^ but there is no standardized survivorship program to enhance health behaviors and/or the psychosocial situation of CAYAs.^3^ Follow-up care is still primarily focused on medical and tumor-specific follow-up.^32,33,34^

The CARE for CAYA-Program (CFC-P) was designed as a multimodal follow-up counseling care program to improve the physical activity, nutritional behavior, and psychosocial needs in CAYAs to reduce the risk for long-term sequelae.

## Methods

### Study Design

This study followed the Consolidated Standards of Reporting Trials (CONSORT) reporting guideline. The CFC-P was an interventional, multicenter, 2-arm parallel group randomized clinical trial (RCT) with a 1:1 allocation and embedded within an ongoing longitudinal cohort study. The study was conducted in adherence to the principles of Good Clinical Practice and the Declaration of Helsinki.^35^ The trial protocol (Supplement 1) was approved by the local ethics committees and published.^3^All participants provided written informed consent; there was no financial compensation.

The CFC-P was conducted in 14 outpatient, mainly university, cancer centers in Germany with needs-based comprehensive assessments to determine high needs in physical activity, nutrition, and psychooncology at baseline (T1) and after 52 weeks (T3). Selected patient self-reported and medical criteria were defined, based on a literature review^3^: (1) for physical activity, insufficient levels of physical activity (screening questionnaire) or criteria of metabolic syndrome; (2) for nutrition, poor dietary quality (assessed with the Healthy Eating Index–European Prospective Investigation Into Cancer and Nutrition [HEI-EPIC]),^36^ underweight (body mass index [BMI]; calculated as weight in kilograms divided by height in meters squared),^37^ gastrointestinal symptoms, or criteria of metabolic syndrome; (3) for psychooncology, symptoms of depression and anxiety (Patient Health Questionnaire-4),^38^ and high distress (National Comprehensive Cancer Network Distress Thermometer)^39^ (eTable 1 in Supplement 2).

If a high need was identified, the intervention group (IG) received 5 counseling sessions, assessments, and newsletters in the respective modules, while the control group (CG) participated in 1 counseling session. The longitudinal cohort study was continued after the RCT to maintain the program and foster the structures at the sites.

### Inclusion Criteria and Randomization

The eligible population included CAYAs aged 15 to 39 years with earlier cancers (*International Statistical Classification of Diseases and Related Health Problems, 10th Revision* codes C00.Z-C97.Z), who had completed acute therapy. Exclusion criteria were severe comorbidities relevant to the module-specific interventions. Data on race and ethnicity were not obtained.

The CAYAs were informed about the CFC-P through regular medical consultations or advertisements at each site. After providing informed consent, the participants were screened and included by the site-related CFC-P staff. Participants with high need in at least 1 module were randomized to IG or CG. A 1:1 randomization was performed by the consortium leadership on request using a concealed, block randomized computer-generated list, stratified by site. The result of the randomization was communicated to the sites by telephone and fax.

After the RCT, the program continued in a longitudinal cohort study. The CAYAs identified with high needs received module-specific interventions. High-need criteria were adapted after the RCT: for physical activity, less than 2 days of intensive physical activity per week; and for nutrition, additionally the self-developed Short Healthy Eating Index Hamburg 2019, with a score of 29 points or less leading to inclusion. The adaption aimed to optimize screening criteria by previously unrecognized needs.

### Study Outcomes

The primary outcome was the rate of CAYAs with high need for at least 1 modular intervention (eTable 1 in Supplement 2) at 52 weeks (T3). A coprimary outcome evaluated the rate of CAYAs with unmet needs, which were outside the scope at week 16 (T2) and T3. Secondary outcomes were feasibility, allocation, and efficacy of modular interventions, QOL, and fatigue (the 30-item European Organisation for Research and Treatment of Cancer Quality of Life [EORTC QLQ-C30] questionnaire)^40^ after 52 weeks (in relation to the initial assessment and the participation in an interventional module), satisfaction (Fragebogen zur Messung der Patientenzufriedenheit [ZUF-8]),^41^ module-specific outcomes (eTable 1 in Supplement 2), and results of the longitudinal study. Further analysis of the co-primary outcome, secondary outcomes cost-effectiveness, and feasibility (process analysis), as well as module-specific outcome, will be published elsewhere.

### Interventions and Assessments

The intervention consisted of additional modular assessments and 5 personalized counseling sessions with each module accompanied by individualized and general newsletters.

In addition to the need-stratified assessment to define high need, assessments via patient-reported outcome (PRO) and medical examination were done at T1, T2, and T3 (eTable 1 in Supplement 2). Participants with no high need were offered 1 counseling session per module. Modular interventions were coordinated with each other and run according to module-specific manuals^3^ (eTable 2 in Supplement 2).

### Statistical Analysis

Data analysis was conducted from August 14, 2021, to May 31, 2022. The sample size calculation for the RCT was performed for the first primary hypothesis with a 2-sided type I error rate of 5% and a power of 90%. Assuming that 90% of CAYAs in the CG still had a high need after 1 year and wanted to show that the intervention reduced this rate by a further 15%, the sample size required for this was calculated as 266. The sample size was increased by 20% to account for potential dropouts. With an assumed rate of survivors without high needs of 40%, 530 CAYAs were calculated for the RCT phase (Supplement 1).

The analysis was based on the full analysis set, which is as close as possible to the intention-to-treat principle. Two primary hypotheses were structured hierarchically: the rate of CAYAs with high need for intervention at T3 is lower in the IG than in the CG, and the rate of CAYAs with unmet needs that are outside the scope of the assessment at T3 is lower than at T2. In case of a nonsignificant primary outcome, the coprimary outcome would be evaluated descriptively. Secondary outcomes were evaluated on an exploratory basis. The 2-sided significance level was set to 5%. For descriptive statistics, categorical data were summarized by absolute and relative frequencies, while continuous data were reported with mean (SD) or median (IQR). The primary hypotheses were tested by comparing the respective rates between the IG and CG with a likelihood-ratio χ^2^ test, and odds ratios (ORs) are reported as effect measures with 95% CIs. Categorical module-specific high need was analyzed in analogy to the first primary hypothesis. Analyses were performed using SPSS, version 27 (IBM Corp),^42^ and R, version 4.0.5 (R Foundation for Statistical Computing).^43^ For the primary outcome, a sensitivity analysis using multiply imputed data (n = 20 imputations) was additionally conducted.

Participants were given 6 weeks to complete the study documents. After that, they were contacted on 3 different days and times of day, and 2 different ways (eg, telephone, e-mail). Missing answers and participant-initiated withdrawals were recorded as a dropout. The RCT data were particularly checked for completeness.

## Results

### Patient Characteristics

**Figure 1.**
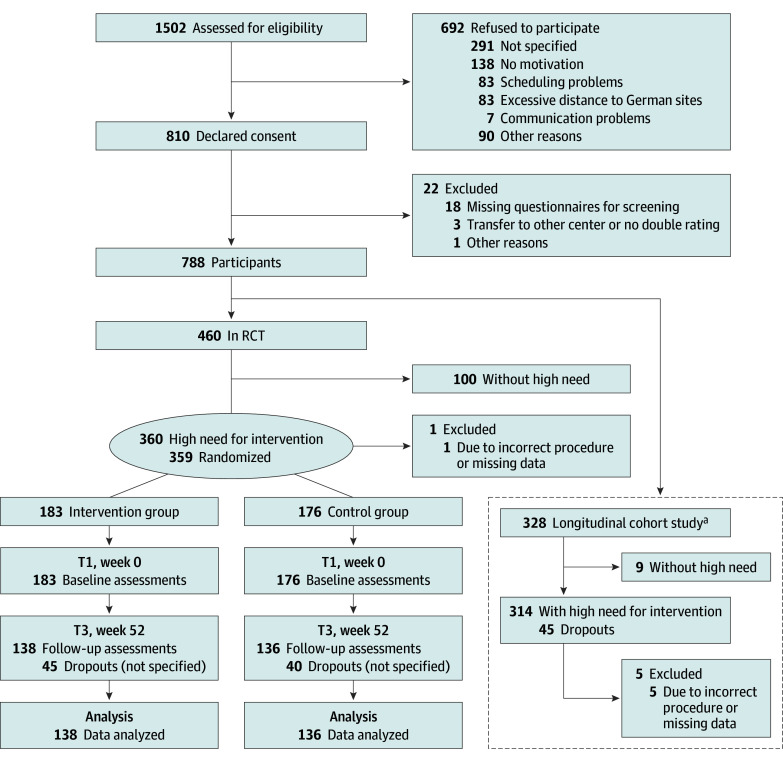
Participant Flowchart Randomized clinical trial (RCT) conducted from to January 1, 2018, to July 15, 2019; longitudinal cohort study conducted from July 16, 2019, to March 31, 2021. ^a^Adapted inclusion criteria, no control group, according to the trial protocol (Supplement 1); results presented elsewhere.^26^

Overall, 1502 CAYAs were screened for eligibility; 692 declined participation, and 788 were included in the CFC-P between January 1, 2018, and November 30, 2020 (Figure 1). Median age was 23.9 (IQR, 19.4-31.1) years, 457 (58.0%) were female, and 331 (42%) were male. The most common diagnoses were lymphoma in 211 (26.8%), sarcoma in 104 (13.2%), and leukemia in 79 (10.0%) participants; 102 individuals (17.9%) had a recurrence of disease or a new disease before study inclusion. The median time since end of tumor therapy was 56.5 (IQR, 12.8-151.0) months. Median BMI was 23.0 (IQR, 20.7-25.9).

**Figure 2.**
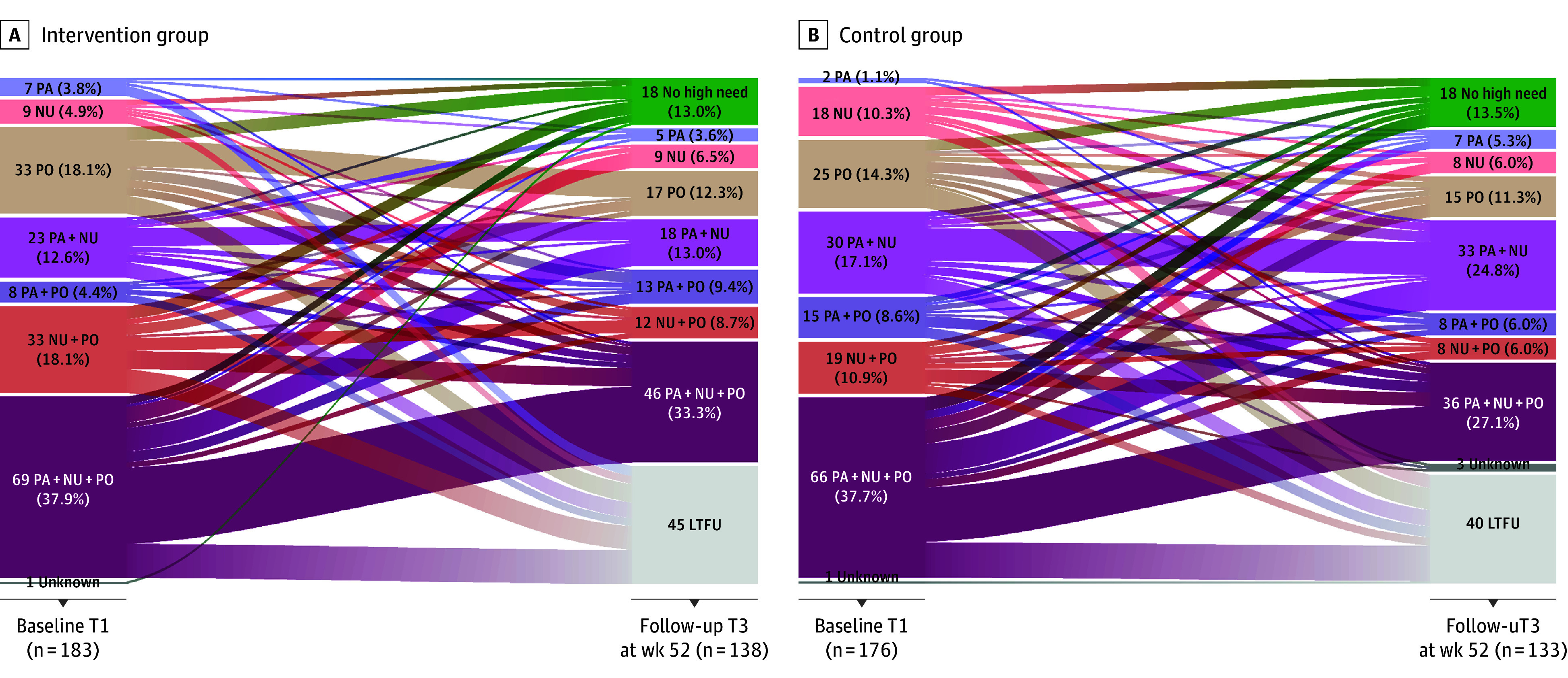
Development of the High Need Cohorts in the Randomized Clinical Trial Data for analysis shown for the intervention (A) and control (B) groups. LTFU indicates lost to follow-up; NU, nutrition; PA, physical activity; and PO, psychooncology.

In the RCT, 460 CAYAs were included between January 1, 2018, and July 15, 2019, with 359 (78.3%) having a high need and being randomized to IG (183 [51.0%]) and CG (176 [49.0%]). The median age was 25.0 (IQR, 19.9-32.2) years; 226 were female (63.0%) and 133 male (37.0%). At T3, data on 274 participants (76.3%) were evaluable, dropout was 45 individuals in the IG and 40 in the CG (23.6%). Most participants had a high need in all 3 modules (Figure 2). Baseline characteristics in the RCT were well balanced between the 2 groups. Participants with no high need were younger (median, 21.7 [IQR, 19.0-27.4] years) and predominately male (66 [60.6%]) (Table 1).

**Table 1.** Patient Characteristics

Characteristic	No. (%)				
	RCT cohort			Longitudinal study cohort	No high need cohort
	Overall	Intervention group	Control group		
Demographic data					
Total participants, No.	359	183	176	788	109
Female	226 (63.0)	111 (60.7)	115 (65.3)	457 (58.0)	43 (39.4)
Male	133 (37.0)	72 (39.3)	61 (34.7)	331 (42.0)	66 (60.6)
Age, median (IQR), y	25.0 (19.9-32.2)	24.8 (20.0-32.1)	25.7 (19.9-32.6)	23.9 (19.4-31.1)	21.7 (19.0-27.4)
Highest school degree					
Total participants, No.	354	181	173	759	98
Current student	38 (10.7)	21 (11.6)	17 (9.8)	96 (12.6)	10 (10.2)
Baccalaureate	153 (43.2)	73 (40.3)	80 (46.2)	344 (45.3)	60 (61.2)
Advanced technical college	41 (11.6)	23 (12.7)	18 (10.4)	78 (10.3)	5 (5.1)
Secondary school leaving certificate	104 (29.4)	54 (29.8)	50 (28.9)	200 (26.4)	19 (19.4)
Elementary or secondary school diploma	13 (3.7)	8 (4.4)	5 (2.9)	31 (4.1)	3 (3.1)
Other	2 (0.6)	2 (1.1)	0 (0.0)	5 (0.7)	1 (1.0)
No school diploma	3 (0.8)	0 (0.0)	3 (1.7)	5 (0.7)	0 (0.0)
Anthropometric data, median (IQR)					
Height, m	1.70 (1.65-1.78)	1.70 (1.65-1.78)	1.70 (1.64-1.76)	1.72 (1.65-1.79)	1.77 (1.69-1.83)
Weight, kg	68.0 (59.0-80.0)	69.8 (61.0-83.0)	67.0 (58.0-75.0)	68.5 (59.0-79.2)	70.0 (62.5-79.3)
BMI	23.4 (20.8-26.6)	23.7 (21.5-27.3)	22.7 (20.3-25.7)	23.0 (20.7-25.9)	22.5 (20.8-24.8)
Tumor-specific data					
Total participants, No.	304	152	152	681	94
Leukemia	39 (10.9)	23 (12.6)	16 (9.1)	79 (10.0)	15 (13.8)
Lymphoma	97 (27.0)	49 (26.8)	48 (27.3)	211 (26.8)	30 (27.5)
Brain tumor	35 (9.7)	14 (7.7)	21 (11.9)	72 (9.1)	2 (1.8)
Sarcoma	45 (12.5)	20 (10.9)	25 (14.2)	104 (13.2)	14 (12.8)
Gastrointestinal tumor	5 (1.4)	2 (1.1)	3 (1.7)	20 (2.5)	5 (4.6)
Urologic tumor	21 (5.8)	11 (6.0)	10 (5.7)	46 (5.8)	8 (7.3)
Gynecologic tumor	19 (5.3)	6 (3.3)	13 (7.4)	46 (5.8)	5 (4.6)
Head and neck tumor	5 (1.4)	5 (2.7)	0 (0.0)	9 (1.1)	2 (1.8)
Thyroid carcinoma	29 (8.1)	17 (9.3)	12 (6.8)	52 (6.6)	6 (5.5)
Other solid tumors	7 (1.9)	4 (2.2)	3 (1.7)	6 (5.5)	2 (1.8)
Skin tumor	2 (0.6)	1 (0.5)	1 (0.6)	1 (0.9)	5 (0.6)
≥1 Recurrence or new disease	47 of 257 (18.3)	28 of 133 (21.1)	19 of 124 (15.3)	102 of 570 (17.9)	8 of 79 (10.1)
Months since therapy, median (IQR)	60.0 (17.0-124.0)	60.5 (16.5-116.8)	58.0 (17.0-132.0)	56.5 (12.8-151.0)	57.0 (14.0-127.5)

Abbreviations: BMI, body mass index (calculated as weight in kilograms divided by height in meters squared); RCT, randomized clinical trial.

In the nonrandomized longitudinal cohort study continued after the RCT, 328 CAYAs with high needs were enrolled and received the interventions from July 16, 2019, to March 31, 2021 (Figure 1). During the overall program, 109 patients with no high needs were included in the following analysis.

### Outcome and Changes of High Needs

#### RCT Results

The rate of CAYAs with at least 1 high need after 52 weeks did not differ significantly between the groups within the IG 87.0% (n = 120) and CG 86.5% (n = 115) (odds ratio [OR], 1.04; 95% CI, 0.51-2.11; *P* = .91). The primary end point was not met. Changes in high needs from T1 to T3 in the each module and the different module combinations are shown in Figure 2 for both groups. A sensitivity analysis with multiply imputed data showed an OR of 0.85 (95% CI, 0.52-1.39; *P* = .52), thus confirming the results of the primary analysis.

Regarding the module-specific changes (Table 2; eTable 3, eTable 7 in Supplement 2), the high need in the module physical activity increased in both groups (OR, 1.52; 95% CI, 0.77-3.08; *P* = .23). Regarding the PRO criteria (identified with screening questionnaire), high needs decreased in both groups from T1 to T3. Concerning the medical criteria (metabolic syndrome), no relevant changes were detected with a persisting high BMI, high waist to hip ratio, and deviating laboratory parameters at T3. In the nutrition module, the high need decreased in both groups with no difference between the IG and CG at T3 (OR, 1.24; 95% CI, 0.69-2.25; *P* = .48). The PRO criteria of poor dietary behavior (Healthy Eating Index-EPIC) improved in both groups from T1 to T3. Regarding the medical criteria, the respective values remained unchanged (low BMI, high BMI or waist to hip ratio, and deviating laboratory test parameters). In the module psychooncology, high needs decreased in both groups, with no difference between the IG and CG at T3 (OR, 1.72; 95% CI, 0.97-3.07; *P* = .06). The PRO criteria anxiety and depression score improved and the distress level decreased from T1 to T3 in both groups. Regarding the coprimary outcome, rate of CAYAs with unmet needs, no significant differences were detected from T2 (12.9% [9 of 70]) to T3 (12.0% [29 of 241]) (OR, 0.93; 95% CI, 0.43-2.17; *P* = .85).

**Table 2.** High Needs in Module-Specific Interventions

Occurrence of high need	Intervention group, No.		Control group, No.	
	T1 (183)	T3 (138)	T1 (176)	T3 (136)
PRO for physical activity, median (IQR)				
Participants, No.	183	138	176	136
Screening questionnaire, moderate activity: d per wk	3.0 (1.0-5.0)	3.0 (2.0-5.0)	3.0 (1.0-5.0)	3.0 (2.0-5.0)
Screening questionnaire, moderate activity: duration, min	45.0 (30.0-67.5)	45.0 (30.0-60.0)	45.0 (30.0-60.0)	45.0 (30.0-60.0)
Screening questionnaire, vigorous activity: d per wk	1.0 (0.0-2.0)	1.0 (0.0-2.3)	1.0 (0.0-2.0)	1.0 (0.0-2.0)
Screening questionnaire, vigorous activity: duration, min	60.0 (30.0-75.0)	45.0 (30.0-60.0)	60.0 (30.0-90.0)	45.0 (30.0-60.0)
PRO for nutrition				
HEI-EPIC [possible range, 0-120]	48.0 (42.0-58.0)	52.0 (42.0-60.0)	48.0 (39.0-59.0)	51.5 (43.0-62.0)
PRO for psychooncology				
NCCN DT score [possible range, 0-10]	6.0 (5.0-8.0)	6.0 (3.0-7.0)	6.0 (4.0-7.0)	5.0 (3.0-7.0)
PHQ-4 score [possible range, 0-12]	3.0 (1.0-6.0)	2.0 (1.0-4.0)	2.0 (1.0-5.0)	1.0 (0.0-3.0)
High need–defining criteria: physical activity, No. (%)				
Participants, No.	107	81	113	89
Questionnaire (PRO)	79 of 106 (74.5)	25 of 79 (31.6)	73 of 110 (66.4)	33 of 82 (40.2)
Metabolic syndrome: BMI	27 of 106 (25.5)	19 of 72 (22.0)	21 of 113 (18.6)	16 of 72 (22.2)
Metabolic syndrome: WHR	23 of 90 (25.6)	15 of 52 (28.8)	27 of 95 (28.4)	13 of 52 (25.0)
Metabolic syndrome: laboratory test results	42 of 95 (44.2)	29 of 59 (49.2)	41 of 96 (42.7)	24 of 53 (45.3)
High need–defining criteria: nutrition				
Participants, No.	134	103	133	104
≤40 HEI-EPIC score (PRO)	26 of 128 (20.3)	16 of 88 (18.2)	37 of 130 (28.5)	9 of 86 (10.5)
BMI <18.5	10 of 133 (7.5)	8 of 89 (9.0)	15 of 132 (11.4)	10 of 89 (11.2)
Gastrointestinal symptoms^a^	38 (31.4)	10 (9.7)	40 (30.1)	10 (9.6)
Metabolic syndrome: BMI	27 of 133 (20.3)	19/ of 9 (21.3)	21 of 132 (15.8)	16 of 89 (18.0)
Metabolic syndrome: WHR	24 of 114 (21.1)	15 of 62 (24.2)	26 of 114 (22.8)	14 of 62 (22.6)
Metabolic syndrome: laboratory test results	43 of 119 (36.1)	29 of 69 (42.0)	40 of 115 (34.8)	25 of 63 (39.7)
High need–defining criteria: psychooncology				
Participants, No.	143	111	125	95
≥6 Points PHQ-4 (PRO)	45 of 141 (31.9)	19 of 108 (17.6)	30 of 125 (24)	12 of 89 (13.5)
≥5 Points NCCN DT (PRO)	137 (95.8)	77 of 109 (70.6)	122 of 124 (98.4)	54 of 89 (60.7)

Abbreviations: BMI, body mass index (calculated as weight in kilograms divided by height in meters squared); HEI-EPIC, Healthy Eating Index–European Prospective Investigation Into Cancer and Nutrition; NCCN DT, National Comprehensive Cancer Network Distress Thermometer; PHQ-4, Patient Health Questionnaire-4; PRO, patient-reported outcomes; T1, baseline; T3, 52 weeks; WHR, waist to hip ratio.

^a^ Only available values.

#### Longitudinal Cohort Study Results

Of 788 participants, 85.4% (n = 673) reported an initial high need. Of these, 475 participants were evaluable at T3; 84.0% (n = 399) continued to report a high need with no significant change (risk difference, −0.84%; 95% CI, −3.32% to 5.02%; *P* = .76). The distribution of high needs was similar in the overall cohort in comparison with the RCT.

### Feasibility of the Overall Study Program

The implementation of the CFC-P at the sites differed regarding time until structures were established (mean, 4.9 months; range, 1-14 months) and first participants included (mean, 6 months; range, 1-10 months). The monthly mean inclusion rate was 23 participants. In the RCT, 274 participants (76.3%) completed the assessment at T3 (Figure 1). The active dropout rate (medical reasons or self-request) was 23.7% (n = 85): 24.6% (n = 45) in the IG and 22.7% (n = 40) in the CG.

Adherence (attending all 5 counseling sessions) was achieved by 63 participants (62.4%) in physical activity, 65 participants (59.6%) in nutrition, and 80 participants (66.1%) in psychooncology. In the CG, 86 participants (68.8%) attended the baseline counseling session in physical activity, 89 participants (70.6%) in nutrition, and 89 (71.8%) in psychooncology (eTable 4 in Supplement 2). A total of 37.6% of the participants showed high needs in all 3 modules and the need-stratified assessment was sufficient, whereby sensitivity could be reconsidered.

### Quality of Life, Fatigue, and Cardiovascular Risk Factors

In the RCT, the QOL score measured with EORTC QLQ-30 changed (T1 to T3) from a median of 74.8 (IQR, 63.3-84.0) to 81.8 (IQR, 67.8-92.3) in the IG and in the CG from 78.8 (IQR, 63.9-87.8) to 85.3 (IQR, 74.1-94.3). The fatigue score decreased in the IG from 55.6 (IQR, 33.3-66.7) to 44.4 (IQR, 22.2-66.7) and in the CG from 44.4 (IQR, 33.3-66.7) to 33.3 (IQR, 22.2-55.6). In the overall cohort, similar results were detected (eTable 5 in Supplement 2). No significant changes in cardiovascular risk factors were observed (eTable 6 in Supplement 2).

### Satisfaction With the CFC-P

**Figure 3.**
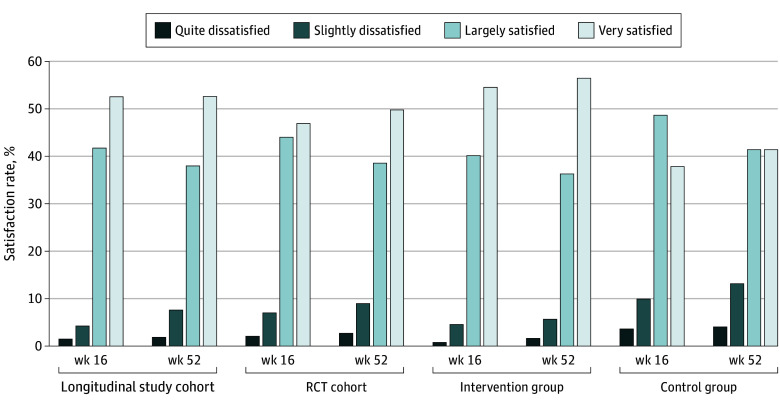
Satisfaction With the CARE for CAYA Program Percentage frequency of each response option for participants' satisfaction with the program in the overall and randomized clinical trial (RCT), longitudinal study, intervention, and control cohorts. CAYA indicates children, adolescents, and younger adults.

In the RCT, 221 participants (90.9%) were satisfied (very and largely satisfied) at T2 and 197 (88.4%) at T3. Satisfaction rate in the IG was higher, with 125 participants (94.7%) at T2 and 115 (92.8%) at T3 compared with the CG, with 96 participants (86.4%) and 82 (82.8%). In the overall cohort, results were similar to the RCT (Figure 3).

## Discussion

To our knowledge, the CFC-P is the first multicenter lifestyle follow-up care program for CAYAs including an RCT and multimodal counseling. The CFC-P confirmed a high need for support in all 3 modules with a rate up to 85% being consistent with previous studies.^5,10,11^ Personalized counseling could not decrease the rate of CAYAs with high needs after 52 weeks or positively influence medical criteria. However, data showed an improvement in the PROs of all participants with increases in self-reported frequency and intensity of physical activity, healthy dietary quality, and anxiety and depression scores. Participants also showed improvements in QOL and fatigue symptoms.

Preliminary studies showed that counseling-based interventions could improve lifestyle behaviors in CAYAs.^24,26^ An enhancement in psychosocial outcomes could be seen in CAYAs who participated in group or individual interventions, reducing symptoms of depression, anxiety, and posttraumatic stress.^44,45^ The need for and the efficacy of need-specific interventions with focus on PROs was also evident in the CFC-P.

However, these effects could not be highlighted with a more intensive approach in the intervention group but were evident for the overall cohort. The multimodal approach may have led to an intervention overload and the selected end point rates of CAYAs with a high need at week 52 might not be specific enough.

Also, the time interval between the end of the intervention at 16 to 24 weeks and measurement of the primary end point at 52 weeks might have been too long. Regarding the physical activity module, counseling alone is estimated to be not sufficient to improve therapy-related long-term effects, but studies have shown that supervised exercise therapy has positive effects on disease-related symptoms.^46^ Furthermore, the so-called Hawthorne effect^47,48^ may have led to an improvement in needs.

### Implications

Although the effect of the interventions was not significant, the personalized approach of the CFC-P to assess the respective individual needs and provide baseline counseling and general newsletters could be easily transferred into survivorship programs for CAYAs. Therefore, a further analysis of who benefits the most and what intervention components are most effective is needed. Because satisfaction with the CFC-P was very high, personalized offers should be further developed in cooperation with CAYAs. The use of internet and peer groups could also lead to improved participant skills outside the CFC-P.^49^ The use of validated instruments for inclusion and outcomes promotes the reliability and validity of the evidence found. In the presence of long-term adverse effects of cancer and its treatment, personalized oncologic exercise and nutritional therapy, as well as psychooncological counseling, remains an important component of medical care.

### Limitations

This study has limitations. The counseling interventions in physical activity, nutrition, and psychooncology were successfully implemented and the outcomes demonstrated the need and feasibility of a supportive program.

The large sample size and inclusion rates indicate that the CFC-P circumvents barriers that have traditionally led to exclusion of CAYAs.^50^ A total of 37.6% of the participants showed high needs in all 3 modules and the need-stratified assessment was sufficient, whereby sensitivity could be reconsidered. The active dropout rate was 23.6%, which is lower than the results of international findings with dropout rates from 30% to 50%.^51,52^

The lack of effect of the overall study program can be partially attributed to the broad definition of the primary end point as well as the complex definition of high need in each module. The median time since the end of tumor therapy was 56.5 months in the longitudinal study. Time since treatment was not an inclusion criterion. This may be a relevant factor in the need for support and possibly for the impact of the program. Further programs should include CAYAs immediately after therapy. The pandemic-related lockdown partly led to a switch to video/telephone contacts, which made it complicated to carry out the interventions as planned. In addition, participating in the CFC-P may have awakened or reawakened needs, being a new or repeat confrontation with cancer and its long-term consequences. This may have also actualized the awareness of better self-care and hereby led the CG to participate in comparative offers. Participation in this survivorship program may already influence coping and disease management. Furthermore, therapy-related reasons for unhealthy lifestyles often cannot be influenced by simply changing a habit and seem to be even more difficult to achieve in the selected population.

## Conclusions

In the RCT of the CFC-P, the complex intervention did not lead to significant changes after 52 weeks, although a high need for support was identified. Overall, patient-reported high needs decreased, although there were no significant between-group differences. A high level of acceptability and satisfaction with the CFC-P was found. Implementation of annual status surveys and need-based, individualized modular interventions may provide optimal supportive care, although the amount, intervention components, and specific target groups need to be finalized.

Counseling interventions regarding physical activity cannot replace supervised exercise therapy and should therefore be offered in addition to or in combination with the exercise intervention. The set of screening tools developed seems to be sufficient to identify high needs not covered by medical follow-up in the CFC-P, and introducing use of the tools in the context of long-term oncologic follow-up may be useful.

## Notes

Supplement 1.
Trial Protocol

Supplement 2. **eTable 1.** Primary Outcome and Secondary Outcomes **eTable 2.** Module-Specific Interventions **eTable 3.** Module-Specific Primary Outcomes and High Need Criteria **eTable 4.** Adherence to Consultations **eTable 5.** Quality of Life and Fatigue (EORTC QLQ-C30) **eTable 6.** Prevalence and Development of Cardiovascular Risk Factors **eTable 7.** Persisting Module-Specific High Need

Supplement 3.
Data Sharing Statement
