# Supplementary material for: A Multimodal Lifestyle Psychosocial Survivorship Program in Young Cancer Survivors: The CARE for CAYA Program—A Randomized Clinical Trial Embedded in a Longitudinal Cohort Study
Source: JAMA Netw Open. 2024 Mar 25;7(3):e242375. doi: 10.1001/jamanetworkopen.2024.2375 (PMC10964114; doi:10.1001/jamanetworkopen.2024.2375)
Supplement: Supplement 1. — Trial Protocol [file jamanetwopen-e242375-s001.pdf]

Supplement 1: The original trial protocol (German)

Titel: "**Comprehensive Assessments and Related interventions to Enhance long-term outcome in Children, Adolescents and Young Adults**"

Das ***CARE for CAYA-Programm***

Präventionsprogramm für junge Patienten nach überstandener Krebserkrankung im Kindes-, Jugend- und jungen Erwachsenenalter (CAYAs)

Ein adaptives und modulares Versorgungsforschungsprogramm

Version 8

Datum: 02.10.2020

Inhaltsverzeichnis

Allgemeine Information **4**

1. **Hintergrund** **7**
   - 1.1 Epidemiologie 7
   - 1.2 Belastungen und Langzeitfolgen 7
   - 1.3 Sport und körperliche Aktivität 9
   - 1.4 Ernährung 9
   - 1.5 Psychosoziales 10
   - 1.6 Fatigue 11
2. **Nachsorge und Interventionen** **12**
   - 2.1 Allgemeine Herausforderungen und aktuelle Versorgungsstruktur 12
   - 2.2 Leben nach Krebs-Programm am UCCH und Interventionsstudien 13
   - 2.3 CARE for CAYA-Programm 14
3. **Hypothesen** **14**
   - 3.1 Primäre Hypothesen 14
   - 3.2 Sekundäre Hypothesen 14
4. **Endpunkte** **15**
   - 4.1 Endpunkte des CARE for CAYA-Programms 15
   - 4.2 Endpunkte der Interventionsmodule 16
5. **Auswahlkriterien** **16**
6. **Studiendesign** **17**
   - 6.1 Organisatorische Struktur des CARE for CAYA-Programms 17
   - 6.2 Ablauf und Methodik des CARE for CAYA-Programms 18
   - 6.3 Zeitliche Abläufe des CARE for CAYA-Programms 27
7. **Statistik** **29**
   - 7.1 Auswertung 29
   - 7.2 Fallzahlkalkulation 31
8. **Datenmanagement** **32**
9. **Abkürzungsverzeichnis** **33**
10. **Literaturverzeichnis** **34**
11. **Anhang** **39**

Zustimmung des Protokolls

Unterschrift des **Studienleiters**:	
Name des Studienleiters:	PD Dr. med. Alexander Stein
Ort und Datum:	Hamburg, den 02.10.2020

Unterschrift des **Methodiker**:	
Name des Methodikers:	Eik Vettorazzi
Ort und Datum:	Hamburg, den 02.10.2020

Allgemeine Information

**Studienleitung**	**PD Dr. med. Alexander Stein**<br>Hubertus Wald Tumorzentrum, Universitäres Cancer Center Hamburg (UCCH), II. Medizinische Klinik und Poliklinik, Universitätsklinikum Hamburg-Eppendorf, Martinistr. 52, 20246 Hamburg
**Studienkoordination/ärztliche und wissenschaftliche Mitarbeiter im UCCH**	**Dr. med. Julia Quidde**<br>**Julia von Grundherr**<br>**Barbara Koch**<br>**Jannike Salchow**<br>Hubertus Wald Tumorzentrum, Universitäres Cancer Center Hamburg, Martinistr. 52, 20246 Hamburg
**Statistik**	**Eik Vettorazzi**<br>Institut für Medizinische Biometrie und Epidemiologie, Universitätsklinikum Hamburg-Eppendorf
**Studienkoordinationspartner am UKE**	**PD Dr. med. Gabriele Escherich**<br>Pädiatrische Hämatologie und Onkologie<br>**Prof. Dr. phil. Corinna Bergelt**<br>Institut und Poliklinik für Medizinische Psychologie
**Studienzentren/Kooperationspartner**	**Prof. Dr. med. Stefan Bielack**<br>Pädiatrie 5 - Onkologie, Hämatologie und Immunologie, Klinikum Stuttgart – Olgahospital, Kriegsbergstraße 62, 70174 Stuttgart<br>**Dr. med. Gabriele Calaminus**<br>Zentrum für Kinderheilkunde, Päd. Hämatologie/ Onkologie, Universitätsklinikum Bonn, Konrad-Adenauer-Allee 119, 53113 Bonn<br>**Prof. Dr. med. Carl Friedrich Classen**<br>Bereich Onkologie und Hämatologie der Universitäts-Kinder- und Jugendklinik Rostock, Universitätsklinikum Rostock, Ernst-Heydemann-Str. 8, 18057 Rostock<br>**Prof. Dr. med. Claudia Rössig**<br>Klinik und Poliklinik für Kinder- und Jugendmedizin, Pädiatrische Hämatologie und Onkologie, Universitätsklinikum Münster, Albert-Schweitzer-Campus 1, 48149 Münster<br>**Univ.-Prof. Dr. med. Jörg Faber**<br>Schwerpunktbereich Pädiatrische Hämatologie/Onkologie-/Hämostaseologie, Universitätsmedizin der Johannes Gutenberg-Universität Mainz, Langenbeckstraße 1, 55131 Mainz<br>**Prof. Dr. med. Peter U. Heuschmann**<br>Institut für Klinische Epidemiologie und Biometrie (IKE-B), Julius-Maximilians-Universität Würzburg, Josef-Schneider-Str. 2 / D7, 97070 Würzburg

	**PD Dr. med. Inken Hilgendorf**<br>Klinik für Innere Medizin II, Abt. für Hämatologie und Internistische Onkologie, Universitätsklinikum Jena, Bachstraße 18, 07743 Jena<br>**Prof. Dr. med. Thorsten Langer**<br>Klinik für Kinder- und Jugendmedizin Pädiatrische Onkologie und Hämatologie, Universitätsklinikum Schleswig-Holstein, Campus Lübeck, Ratzeburger Allee 160, 23538 Lübeck<br>**Prof. Dr. med. Markus Metzler**<br>Abteilung für Pädiatrische Onkologie und Hämatologie der Kinder- und Jugendklinik, Universitätsklinikum Erlangen, Loschgestraße 15, 91054 Erlangen<br>**Prof. Dr. med. Charlotte Niemeyer**<br>Tumorzentrum Freiburg – CCCF, Robert-Koch-Klinik, EG Universitätsklinikum Freiburg, Hugstetter Straße 55, 79106 Freiburg<br>**Prof. Dr. med. Dirk Reinhardt**<br>**Prof. Dr. med. Uta Dirksen**<br>Klinik für Kinderheilkunde III, Universitätsklinikum Essen, Hufelandstraße 55, 45122 Essen<br>**Dr. med. Annette Sander**<br>Pädiatrische Hämatologie und Onkologie, Medizinische Hochschule Hannover, Carl-Neuberg-Str. 1, 30625 Hannover<br>**Dr. rer. medic. Michael Köhler**<br>Arbeitsbereich Psychoonkologie, Universitätsklinik für Hämatologie und Onkologie, Leipziger Straße 44, 39120 Magdeburg<br>**Prof. Dr. med Jens Habermann**<br>Universität zu Lübeck, Interdisziplinäres Centrum für Biobanking-Lübeck (ICB-L), Ratzeburger Allee 160, 23562 Lübeck
**Modulverantwortliche/wissenschaftliche Studienleitung**	**Ernährung:**<br>Julia von Grundherr, Barbara Koch, Julia Quidde<br>**Sport und körperliche Aktivität:**<br>Jannike Salchow, Dr. Sportwiss. Wiebke Jensen, Barbara Koch, Julia Quidde<br>**Spiroergometrie**<br>Prof. Dr. med. Rüdiger Reer, UKE Sportpraeventicum<br>**Psychoonkologie:**<br>Prof. Dr. phil. Corinna Bergelt<br>Dr. phil. Frank Schulz-Kindermann<br>**Neurokognition:**<br>PD Dr. Gabriele Escherich, Barbara Koch, Tanja Tischler<br>**Lebensqualität, Fatigue:**<br>Dr. Gabriele Calaminus<br>**Metabolisches Syndrom:**<br>Prof. Dr. Thorsten Langer, Prof. Dr. Brabant

**Advisory Board Members**	**Prof. Dr. Luzia Valentini**<br>Fachbereich Agrarwirtschaft und Lebensmitteltechnologie, Hochschule Neubrandenburg - University of Applied Sciences, Neubrandenburg<br>**PD Dr. Freerk Baumann**<br>Leiter AG Onkologische Bewegungsmedizin, Centrum für Integrierte Onkologie Köln Bonn, Universitätsklinikum Köln<br>**Prof. Dr. Susanne Singer**<br>Leiterin der Abteilung Epidemiologie und Versorgungsforschung an der Universitätsmedizin Mainz<br>**Prof. Dr. Holger Schulz**<br>Stiftungslehrstuhl Klinische Versorgungsforschung am Institut für Medizinische Psychologie, Universitätsklinikum Hamburg-Eppendorf

1. Hintergrund

1.1 Epidemiologie

Jedes Jahr erkranken in Deutschland insgesamt rund 480.000 Menschen an Krebs. Die Zahl der Neuerkrankungen hat zwischen 2002 und 2012 bei Männern um 13% und bei Frauen um 10% zugenommen [1]. Die Anzahl der Krebsüberlebenden wird laut Robert Koch Institut gegenwärtig auf 2,6 Millionen geschätzt (vorläufige 10-Jahres-Prävalenz für 2012) und steigt durch Fortschritte in der Diagnostik und Therapie von Krebserkrankungen stetig an.

Junge Patienten nach überstandener Krebserkrankung im Kindes-, Jugend- und jungen Erwachsenenalter werden weltweit unter dem Begriff „CAYA“ (Children, Adolescents and Young Adults) zusammengefasst. Dabei sind in der Literatur unterschiedliche Altersgrenzen beschrieben, wobei die häufigste untere Altersgrenze zwischen 15 und 18 Jahren und die oberste Grenze mit 39 Jahren angegeben wird (DGHO 2015 [2], NCCN Guideline [3]).

Laut der Datenbank des Zentrums für Krebsregisterdaten (ZfKD)[1] des Robert-Koch-Institutes erkrankten in der Altersgruppe von 15 bis 39 Jahren im Jahr 2012 insgesamt 8.985 Frauen und 6.282 Männer neu an Krebs. Die häufigsten onkologischen Neuerkrankungen von Frauen in der Altersgruppe 15-19 Jahre sind das Hodgkin Lymphom, in der Altersgruppe 20-29 Jahre das Melanom und in der Altersgruppe 30-39 Jahre das Mammakarzinom. Bei Männern sind in allen Altersgruppen von 15-39 Jahren Keimzelltumoren die häufigste onkologische Neuerkrankung [4].

Die Anzahl der Neuerkrankungen bei Kindern unter 15 Jahren liegt bei jährlich etwa 1 .800 Fällen. Das Diagnosespektrum bei Kindern unterscheidet sich grundsätzlich zu dem der Erwachsenen. Die häufigsten Krebserkrankungen bei Kindern stellen die Leukämien (33,4%), die ZNS-Tumoren (24,4%) und die Lymphome (10,9%) dar. Bei 4,4% wird innerhalb von 25 Jahren mindestens eine weitere Krebserkrankung diagnostiziert [1].

Die Behandlung von Krebserkrankungen bei Kindern und Jugendlichen hat in den letzten Jahrzehnten erhebliche Fortschritte gemacht. Die 5-Jahres-Überlebensraten für maligne Erkrankungen im Kindes- und Jugendalter sind von weniger als 10% bis 20% in den 50er- und 60er-Jahren auf über 75% angestiegen [5]. Bei Patienten mit Morbus Hodgkin oder Retinoblastom liegt die 5-Jahres-Überlebensrate bei 90%. Bei der akuten lymphatischen Leukämie (ALL) und bei Non-Hodgkin-Lymphomen (NHL) ist die 5-Jahres-Überlebensrate mit über 80% heute weit höher als noch vor 50 Jahren [5]. Die Überlebenswahrscheinlichkeit für erkrankte Kinder unter 15 Jahren liegt mittlerweile bei 84% nach 5 Jahren, bei 82% nach 10 Jahren und 81% nach 15 Jahren [1]. Somit steigt, einhergehend mit den verbesserten Behandlungsmöglichkeiten, die Anzahl der CAYA-Krebsüberlebenden stetig an [6-8].

a. Belastungen und Langzeitfolgen

Multimodale Therapiekonzepte, die lokale Therapieverfahren wie Operationen und Strahlentherapie mit systemischen Therapien kombinieren, eröffnen einer zunehmenden Zahl an Patienten mit hämatologischen oder onkologischen Malignomen die Chance auf Heilung. Diese komplexen Therapiestrategien vergrößern allerdings nicht nur die Heilungs- und Überlebenschancen, sondern können auch zum vermehrten und verstärkten Auftreten von Akut- und Langzeitnebenwirkungen führen. In einer

---

[1] http://www.krebsdaten.de/Krebs/DE/Datenbankabfrage/datenbankabfrage_stufe1_node.html, Abgerufen am 26.10.2016.

groß angelegten Kohortenstudie (Childhood Cancer Survivor Study) an 10.397 Patienten zeigten Oeffinger et al., dass etwa zwei Drittel der CAYAs unter den therapiebedingten Folgen leiden [9]. Dazu gehören sowohl körperliche Probleme z.B. Polyneuropathie, chronische Schmerzen, Erschöpfungszustände, Herzkreislauferkrankungen [10-13], als auch psychische Beschwerden z.B. Rezidiv- oder Zukunftsängste, Depressionen, Anpassungsstörungen [6, 14-16], weiterhin soziale Probleme z.B. Abbruch der Schul- oder Berufsausbildung, ausbleibende Reintegration in die Arbeitswelt, finanzielle Schwierigkeiten [6, 17] sowie kognitive oder neurologische Beeinträchtigungen wie z.B. Konzentrations- oder Gedächtnisstörungen [18, 19]. Darüber hinaus besteht neben dem Risiko für ein Wiederauftreten der Tumorerkrankung auch ein lebenslang erhöhtes Risiko für Zweitmalignome. Dabei ist das Intervall bis zum Auftreten von hämatologischen Neoplasien kürzer als das von soliden Tumorerkrankungen [20].

Zu den häufigsten Langzeitfolgen nach antineoplastischen Therapien im Kindes-, Jugend- und jungen Erwachsenenalter gehören die kardiovaskulären Erkrankungen, die ebenfalls die häufigste nichtmaligne Todesursache der CAYAs repräsentieren [21, 22]. Im Vergleich zu der Normalbevölkerung ist das Risiko, eine Herzkreislauferkrankung zu entwickeln je nach Risikoprofil 5 bis 15-fach erhöht [9, 11-13, 21, 23], dabei tragen Leukämie- und Brustkrebsüberlebende ein besonders hohes Risiko für eine kardiovaskuläre Erkrankung [23]. Für dieses erhöhte Risiko sind sowohl therapiebedingte (u.a. Art der Chemotherapie und deren Infusionsdauer, Applikationsart, Gesamtdosis und Anzahl der Zyklen, Bestrahlung im Bereich des Brustkorbs) als auch allgemeine kardiovaskuläre Faktoren wie z.B. Hypertonie, Hyperlipidämie, Adipositas, Alter, kardiovaskuläre Vorerkrankungen, familiäre Komponenten und Geschlecht verantwortlich [24].

Im Rahmen von Therapieoptimierungsstudien wird seit geraumer Zeit versucht die Langzeitfolgen von Krebstherapien zu minimieren z.B. durch risikoadaptierte Therapiestrategien, durch Dosisanpassungen, Präparatewechsel und Optimierung der Therapiestrategien (z.B. Strahlentherapie nach Chemotherapie nur noch bei einem bestimmten Teil der Patienten mit verbliebenen Tumorrest und Aktivitätszeichen in der Bildgebung). Allerdings ist dies aufgrund der zwingend zu erhaltenden hohen Heilungsraten nur in begrenztem Ausmaß möglich. So müssen einige Medikamente bzw. Therapiebestandteile mit bekannten Langzeitfolgen in Ermangelung von gleich wirksamen aber besser verträglichen Alternativen beibehalten werden.

Im Gegensatz dazu interagieren Maßnahmen zur Verbesserung des Lebensstils nicht mit den jeweiligen Krebstherapiekonzepten oder Heilungsraten, haben aber nachweislich einen positiven Effekt auf Langzeitfolgen. So wurde in Studien bei Erwachsenen Krebsüberlebenden gezeigt, dass eine gesunde und ausgewogene Ernährung sowie eine regelmäßige körperliche Aktivität das Risiko für Folgeerkrankungen wie Herz-Kreislauferkrankungen oder Übergewicht reduzieren können. Zudem wirkt sich ein gesunder Lebensstil positiv auf das psychische Wohlbefinden und die Lebensqualität aus [25-28].

Durch Langzeitfolgen können kostenintensive Folgebehandlungen notwendig werden [29, 30]. Nicht zuletzt kann die Krebserkrankung eine finanzielle Belastung für Krebsüberlebenden mit sich bringen [29]. Die Rückkehr in Ausbildung und Arbeitswelt nach Abschluss der Therapie kann verzögert, nur in geringerem Ausmaß oder nicht möglich sein, zudem kann es zu Produktivitätsausfällen und vermehrten Krankheitstagen kommen, dadurch resultiert bei den betroffenen Krebsüberlebenden zumeist ein geringeres Einkommen [29, 31-34]. Diese Einbußen an Einkommen können sich auf einen guten und gesunden Lebensstil auswirken. Um direkte und indirekte Krankheitskosten abschätzen zu können, wird die Inanspruchnahme von medizinischen Leistungen und Behandlungen erhoben. Durch das CARE for CAYA-Programm sollen kostenintensive Langzeitfolgen vermieden oder minimiert und damit direkte und indirekte Krankheitskosten reduziert werden. Zudem soll die Teilhabe an Ausbildung und Arbeitsleben verbessert werden.

b. Sport und körperliche Aktivität

Zahlreiche Studien haben die Bedeutung von Sport und körperlicher Aktivität bei Krebspatienten untersucht. Diese Studien zeigten, dass durch regelmäßige körperliche Aktivität krankheits- und therapiebedingte Symptome verbessert werden können. Sportliche Aktivität wirkt sich positiv auf die kardiovaskuläre Fitness, Muskelkraft, Körperzusammensetzung, Angst, Depressionen, psychosoziale Beschwerden und Belastungen, Selbstwertgefühl und Lebensqualität von Krebsüberlebenden aus. Darüber hinaus gibt es für einige Tumorarten Hinweise auf ein geringeres Rezidivrisiko bei körperlich Aktiven [25, 35]. Therapiebedingte Langzeitfolgen können durch Sport verbessert werden, u.a. zeigt sich eine positive Wirkung auf die Fatigue-Symptomatik [36, 37].

Aus dem Bereich der Kardiologie ist bekannt, dass regelmäßige körperliche Aktivität einen präventiven Einfluss auf die Entstehung und Ausprägung von koronaren Herzkrankheiten (KHK) hat [26, 38-41]. Eine retrospektive Kohortenstudie bei Langzeitüberlebenden nach Hodgkin Lymphom zeigte, dass anstrengende körperliche Aktivität mit mehr als 9 metabolisches Äquivalent (MET) pro Woche, das Risiko für therapiebedingte kardiovaskuläre Erkrankungen verringert [28].

CAYAs ohne Empfehlungen zur körperlichen Aktivität, haben größere Probleme das ehemalige Bewegungsverhalten zu erreichen als CAYAs mit entsprechenden Empfehlungen oder Beratungen [42]. In der Beratung zu Sport und körperlicher Aktivität müssen Bewegungseinschränkungen sowie physische Stärken und Schwächen beachtet und individuelle Ziele und Vorlieben berücksichtigt werden [43].

Die American Cancer Society sowie das American College of Sports Medicine empfehlen Krebsüberlebenden sich pro Woche 150 Minuten mit moderater Intensität oder 75 Minuten mit intensiver Intensität körperlich zu betätigen und bewegungsarme, sitzende Tätigkeiten zu vermeiden oder zu reduzieren [25, 35, 44]. Die körperliche Aktivität sollte mindestens 10 Minuten am Stück ausgeübt werden. Darüber hinaus sollten Erwachsene mindestens zweimal die Woche Kräftigungsübungen der großen Muskelgruppen ausüben.

Insgesamt liegen für die Gruppe der CAYAs zu wenige Daten über die Effekte körperlicher Aktivität auf Langzeitfolgen nach einer Krebserkrankung und Therapie vor.

c. Ernährung

Das Gesundheits- und Ernährungsverhalten vieler Krebsüberlebender weist Defizite auf. Nur 10% der Krebsüberlebenden geben an, einen gesunden Lebensstil und Normalgewicht zu haben. 58% der Krebsüberlebenden sind übergewichtig, 55% treiben nur selten Sport und 82% essen weniger als die empfohlenen 5 Einheiten Obst oder Gemüse täglich [45]. Auch die INAYA-Studie (Improved Nutrition in AYAs) zeigte, dass nur 21,7% der CAYAs ein gutes Ernährungsverhalten aufweisen. 73,9% und 4,3% der CAYAs hatten ein verbesserungswürdiges oder schlechtes Ernährungsverhalten [46].

Eine gesunde Ernährung bei Krebsüberlebenden kann zur Senkung des kardiovaskulären Risikos führen [26, 27, 47]. Beispielsweise kann eine mediterrane Ernährungsform das Risiko für ein kardiovaskuläres Ereignis um 30% senken [48].

Für die Prävention von onkologischen Ereignissen zeigten Studien, dass insbesondere ein hoher Konsum von rotem Fleisch, zum Beispiel Rind- oder Schweinefleisch, mit einem erhöhten Risiko für Kolon- und Mammakarzinome sowie Gesamttumormortalität verbunden ist [49-51]. In Ergänzung dazu empfiehlt der WCRF (World Cancer Research Fund) den Verzehr von energiehaltigen Lebensmitteln und zuckerreichen Getränken zu begrenzen, dagegen überwiegend pflanzliche Lebensmittel im Rahmen einer obst-, gemüse-, ballaststoffreichen Ernährung zu verzehren, den Verzehr von verarbeitetem Fleisch zu vermeiden, eine Begrenzung des Konsums von alkoholischen Getränken, eine Begrenzung

des Salzkonsums und den Verzicht auf Nahrungsergänzungsmittel [52, 53]. Strukturierte Schulungs- und Behandlungsprogramme über den Stellenwert der Ernährung bei KHK führen zum einen zu einer Verbesserung des Ernährungsmusters und zum anderen zu einer Steigerung der Lebensqualität von KHK-Patienten [54]. Darüber hinaus zeigten Studien, dass Ernährungsinterventionen zur Senkung des kardiovaskulären Risikos effektiv sind [55, 56].

Dass die Ernährung auch bei Krebspatienten unter und nach Therapie wichtig ist, wurde ebenfalls in diversen Studien gezeigt. Der Review „Counseling to Promote a Healthy Diet“ aus dem Jahr 2002 analysierte mittels systematischer Übersichtsarbeiten, Beobachtungsstudien und randomisierten Studien den Zusammenhang von Ernährungsgewohnheiten und deren gesundheitlichen Folgen. Hierbei konnte in mehreren Studien gezeigt werden, dass durch eine Beratung das Ernährungsverhalten der Patienten, insbesondere durch Reduktion der allgemeinen Fettaufnahme und von gesättigten Fettsäuren sowie die Erhöhung des Obst- und Gemüseverzehrs, verbessert werden konnte [55].

Nach den Empfehlungen des WCRF sollten Krebsbetroffene und damit auch Krebsüberlebende eine Ernährungsintervention, in diesem Fall in Form von Ernährungsempfehlungen, erhalten [52, 53]. Die INAYA-Studie zeigte, dass nur ein Fünftel der Patienten Vorerfahrungen mit Ernährungsberatungen hatten und auch die Querschnittsstudie von N. Bader et al. mit 236 Brustkrebsüberlebenden konnte zeigen, dass nur 27,1% der Patientinnen eine Ernährungsberatung während und/oder nach der Therapie angeboten bekommen hatten [57]. Diese Ergebnisse zeigen, dass eine zu geringe Anzahl von Krebsüberlebenden eine Ernährungsberatung erhält, welche jedoch aufgrund des verbesserungswürdigen Ernährungsverhaltens und des Risikoprofils dieser Patientengruppe anzuraten ist.

In der INAYA 1 und 2 Studie des Universitären Cancer Centers Hamburg berichteten viele Patienten nach überstandener Krebserkrankung von Geschmacksveränderungen, u.a. wurde deutlich, dass ein Großteil der Patienten zu viel Salz zu sich nahm [46]. Geschmacksbeeinträchtigungen können sowohl als akute Nebenwirkungen unter laufender Therapie als auch viele Jahre danach als Langzeitfolgen der Therapie auftreten [58]. Zur weiteren Differenzierung und Optimierung des Ernährungsverhaltens wird der Geschmacksinn untersucht.

d. Psychosoziales

Eine Krebserkrankung im Kindes- und Jugendalter wirkt sich auf die körperliche, soziale, emotionale oder kognitive Entwicklung des Jugendlichen aus und verdoppelt das Risiko, Verhaltensauffälligkeiten oder Schulprobleme zu entwickeln [59]. Die Ergebnisse von Studien zu Spät- und Langzeitfolgen für Kinder und Jugendliche, die an Krebs erkrankt waren, verdeutlichen, dass diese nicht nur unter z.T. irreversiblen körperlichen Auswirkungen und Schädigungen wie z.B. Wachstums- oder Fertilitätsstörungen leiden [60], sondern zudem eine geringere Lebenszufriedenheit als die Normalbevölkerung aufweisen [61]. Kinder und Jugendliche, die eine Krebserkrankung überleben, konnten unter Therapie häufig für lange Zeit nicht ihrem normalen Alltag nachgehen, beispielsweise mussten sie aufgrund von Infektionsgefahren über mehrere Monate ohne Kontakt zu (gleichaltrigen) Kindern leben.

Studien zeigen, dass eine Krebserkrankung, die Behandlung und deren Folgen bei Kindern und Jugendlichen zu geringerer Lebensqualität [62] und vermindertem psychischem Wohlbefinden [63] führen können. Auch Verhaltensauffälligkeiten und Schwierigkeiten bei der Reintegration in die Schule können auftreten [64]. Die Rückkehr in den Alltag und das Berufsleben bei jungen Erwachsenen ist häufig erschwert und nur langsam umsetzbar [14], so sind in einer Analyse 21% der berufstätigen Krebsüberlebenden ein Jahr nach Rehabilitationsmaßnahmen nicht in den Beruf zurückgekehrt [65]. Neben körperlichen Folgeschäden wie Störungen im Wachstum, der Pubertätsentwicklung oder Fertilität [66], können Patienten noch lange nach Abschluss der Behandlung an erhöhtem Distress leiden [67] und

haben ein größeres Risiko für die Entwicklung einer psychischen Störung [68]. Krebsüberlebende weisen spezifische Bedürfnisse auf, die allerdings bei der Hälfte der Patienten unerfüllt bleiben und wesentlich mit der Ausprägung von Angst vor Tumorrezidiven korrelieren können [69, 70].

Der durchschnittlich moderate korrelative Zusammenhang zwischen psychischer Belastungen und den subjektiven Unterstützungsbedürfnissen verdeutlicht, dass der Unterstützungsbedarf von krebskranken Menschen nicht unmittelbar aus dem Ausmaß an psychischer Belastung abgeleitet werden kann [71]. Aus diesem Grund sieht das psychosoziale Modul des *CARE for CAYA-Programms* neben einer psychoonkologisch fundierten Einzelintervention (*Motivational Interviewing*), welche sich spezifisch an die Subgruppe der hochbelasteten jungen Patienten richtet, vor, dass alle CAYAs, unabhängig von der im Screening ermittelten psychischen Belastung, bei Bedarf die an den teilnehmenden Standorten verfügbaren psychoonkologischen Versorgungsangebote in Anspruch nehmen können.

Motivational Interviewing ist ein sowohl klientenzentrierter als auch direktiver Ansatz der therapeutischen Gesprächsführung mit dem primären Ziel der Erhöhung der Eigenmotivation, ein persönlich relevantes Ziel durch Verhaltensveränderung selbstwirksam zu erreichen [72, 73]. Die Grundannahme des *Motivational Interviewing* ist das Ambivalenzmodell, d.h. Menschen sind nicht unmotiviert oder unfähig, wenn es ihnen nicht gelingt ein Problemverhalten zu ändern bzw. ein persönliches Ziel zu verfolgen, sondern sie sind ambivalent. Die Motivationsarbeit mit CAYAs orientiert sich demzufolge primär an der Exploration und Reduzierung der motivationalen Ambivalenzen des Patienten.

Folgende vier Prinzipien finden beim *Motivational Interviewing* Anwendung:

1) *Empathie* – zentrale Grundlage ist das Bestreben dem Patienten respektvoll zuzuhören und sein Erleben zu verstehen sowie zu akzeptieren.

2) *Entwicklung von Diskrepanzen* – im Prozess soll dem Patienten die motivationale Zwiespältigkeit erlebbar gemacht werden und die Wichtigkeit einer Veränderung soll an innerer Kraft gewinnen.

3) *Umgang mit Widerstand* – um Widerstand gegen eine Veränderung zu verhindern, wird der Patient nicht zu etwas bewogen, sondern in seinem Widerstand wertgeschätzt und verstanden.

4) *Stärkung der Veränderungszuversicht* – Erhöhung der Selbstwirksamkeitserwartung und der Zuversicht, ein Verhalten ändern zu können.

Motivational Interviewing fand bereits vereinzelt Anwendung im Bereich der Psychoonkologie mit dem Ergebnis, dass *Motivational Interviewing* effektiv ist hinsichtlich gesundheitsfördernden Verhaltens als auch psychosozialer Unterstützungsbedürfnisse von Krebspatienten [74].

e. Fatigue

Bei vielen Krebsüberlebenden gehört Fatigue mit zu den problematischsten Langzeitfolgen. Unter Fatigue versteht man eine außerordentliche Müdigkeit, mangelnde Energiereserven oder ein massiv erhöhtes Ruhebedürfnis, das absolut unverhältnismäßig zu vorangegangenen Aktivitätsänderungen ist und sich durch normale Erholungsmechanismen (z.B. Schlaf) nicht beheben lässt.

Fatigue kann zu jedem Zeitpunkt unter und nach der Erkrankung und Therapie entstehen. Nicht selten hält die Fatigue über das Behandlungsende hinaus an oder entsteht erst nach Beendigung der Behandlung. Je nach Studie leiden 33-60% der Langzeitüberlebenden 5 Jahre nach Therapieende noch an Fatigue [75-77], was sich auf die Alltagsgestaltung und Re-Integration der Betroffenen negativ auswirken kann [77].

Fatigue als multifaktoriellen Geschehen, kann in allen Altersskalen auftreten. In einer qualitativen Studie beschrieben Kinder und Jugendliche „ein ständiges Bedürfnis sich hinzulegen“, „einfach gar nichts

tun zu wollen“, „nicht normal zu sein“ und bemerkten dass sich dieses Verhalten von ihrem normalen Aktivitätslevel unterschied [78].

In unterschiedlichen Altersbereichen können unterschiedliche Aspekte von Fatigue im Vordergrund stehen. So zeigten Studien, dass onkologisch erkrankte Kinder Fatigue stärker als verringerte körperliche Leistungsfähigkeit wahrnehmen und andere Aspekte aufgrund des Alters noch nicht reflektiert werden können. Onkologisch erkrankte Jugendliche und junge Erwachsene nahmen hingegen zusätzlich zur körperlichen Fatigue auch die emotionale und mentale Fatigue wahr und ordneten diese als Folge der Erkrankung und/oder Behandlung ein [79, 80]. Edwards et al. (2003) schlussfolgerten in einer Zusammenfassung dementsprechend, dass das Erleben von Fatigue mit der kognitiven Entwicklung konfundiert ist [76].

2. Nachsorge und Interventionen

1.1 Allgemeine Herausforderungen und aktuelle Versorgungsstruktur

Die Versorgungsstruktur der CAYA-Patienten ist sehr heterogen. Patienten bis einschließlich des 18. Lebensjahres, teilweise aber auch deutlich länger, werden durch pädiatrische Fachärzte, meist an spezialisierten Zentren, betreut. Die über 18-jährigen Patienten werden durch verschiedene Fachdisziplinen betreut z.B. Hämatologie/Onkologie, Gynäkologie oder Dermatologie. Der Übergang (Transition) der Patienten bei Überschreitung des 18. Lebensjahres ist von entscheidender Bedeutung, da es durch den Abbruch der kontinuierlichen Betreuung zu einem Verlust wichtiger patientenspezifischer Informationen kommen kann.

Darüber hinaus ist die Durchführung der Nachsorge sehr unterschiedlich. Der überwiegende Teil der pädiatrischen Patienten wird im Rahmen von Studien oder Registern therapiert und wird entsprechend den Vorgaben der jeweiligen Studien- oder Registerempfehlungen nachgesorgt. Bei den über 18-jährigen Patienten kommen jedoch häufig allgemeine Nachsorgeempfehlungen zum Einsatz, die für die Gesamtheit der erwachsenen Patienten dieses Kollektivs angewandt werden und meist nur auf Detektion von Rezidiven ausgelegt sind. Bei weit mehr als der Hälfte der Krebsüberlebenden wird der bestehende Unterstützungsbedarf insbesondere in körperlichen oder psychosozialen Bereichen häufig gar nicht erst erhoben und bleibt demnach unerfüllt [16, 81, 82].

Die aktuelle Versorgungsstruktur der CAYAs bezieht sich zumeist auf die medizinische Tumornachsorge und beinhaltet in der Regel kaum präventive-Maßnahmen oder Interventionsangebote in den genannten Bereichen. Diese Nachsorge wird bislang zumeist rein ärztlich durchgeführt, obwohl in nationalen und internationalen Empfehlungen und Leitlinien die Nachsorge im Rahmen von multiprofessionelle Teams empfohlen wird, um die Bereiche Ernährung, Sport und Psychosoziales präventiv zu adressieren [25, 43, 52, 83-86].

Um den individuellen Bedarf und der Prävention von Therapiefolgen gerecht werden zu können, bedarf es neben der medizinischen Tumornachsorge und/oder der Behandlung von symptomatischen Therapiefolgen einer kontinuierlichen, multiprofessionellen und interdisziplinären Betreuung durch Ärzte und Spezialisten verschiedener medizinischer und nicht-medizinischer Fachdisziplinen, wie z.B. Hämatologen und Onkologen der Kinderheilkunde und Erwachsenenmedizin, Sporttherapeuten, Ernährungsberater, Psychoonkologen und Sozialarbeiter.

Zentrale Aspekte der Beratung sollten Empfehlungen hinsichtlich körperlicher Aktivität und Ernährung, allgemeine Empfehlungen zum Lebensstil und Gesundheitsverhalten u.a. Rauchen und Alkoholkonsum, Gespräche über psychische Belastungen, ggf. Themen wie Freunde, Familie, Beziehungen, sexuelle Gesundheit, Fertilität, Schule, Arbeit, Zukunftsplanungen und Fatigue, Angst und Depressionen beinhalten [87-89].

Das gesamte Nachsorgeprogramm der CAYAs sollte demnach individuell auf den Patienten zugeschnitten sein.

a. Leben nach Krebs-Programm am UCCH und Interventionsstudien

Im Jahr 2012 wurde am Universitären Cancer Center Hamburg das *Leben nach Krebs-Programm* implementiert, das zum einen die Spezialsprechstunde für Erwachsene (>40 Jahre) nach überstandener Krebserkrankung, zum anderen die interdisziplinäre pädiatrisch-internistische CAYA-Sprechstunde für Jugendliche und junge Erwachsene (15-39 Jahre) nach überstandener Krebserkrankung im *CARE for CAYA-Programm* umfasst. Ziele des *Leben nach Krebs-Programms* sind eine leitlinienbasierte und individualisierte Nachsorge zum frühzeitigen Erkennen eines Rezidives der initialen Tumorerkrankung, die Vorsorge zum frühzeitigen Erkennen von Langzeitfolgen der Tumortherapie oder Tumorerkrankung und ggf. deren Behandlung sowie die Prävention sekundärer Erkrankungen.

Darüber hinaus werden alle CAYAs, die sich im Rahmen des *Leben nach Krebs-Programms* des UCCH vorstellen, hinsichtlich ihres Lebensstils beraten. Im derzeitigen Fokus stehen dabei die Beratung zu Sport und körperlicher Aktivität durch eine Sportwissenschaftlerin sowie die Ernährungsberatung durch eine Diätassistentin/ Ernährungswissenschaftlerin.

Im Rahmen *des Leben* nach Krebs-Programms wurden und werden bereits monozentrische Studienprojekte zum Thema Ernährung und Sport durchgeführt.

In der Pilotstudie INAYA 1 (**„I**mproved **N**utrition in **AYA**s**“ – INAYA 1**, DRKS-ID 00009883) wurde gezeigt, dass das Ernährungsverhalten von CAYAs durch eine intensivierte Ernährungsberatung relevant verbessert werden kann [46]. Bereits nach 3 Monaten ergab sich eine deutliche Verbesserung der Rate an CAYAs mit gutem Ernährungsverhalten von 4% auf fast 50% und ein Rückgang der CAYAs mit schlechtem Ernährungsverhalten von 22% auf 0% [46, 90].

In der Nachfolgestudie INAYA2 (**„I**mproved **N**utrition in **AYA**s with Reduction of Sodium Intake**”** - **INAYA 2**, DRKS00010695) wurde bei der Zwischenauswertung der ersten 20 Patienten eine signifikante Reduktion des Salzkonsums gemäß der Ernährungsprotokolle gesehen. Nach 3 Monaten sank der mediane tägliche Salzkonsum hierbei von 8,58 g auf 6,96 g (p= 0,044).

In einer ersten Zwischenauswertung der randomisierten Sportberatungsstudie MAYA (**„M**otivate **AYA“/MAYA**, DRKS-ID 00009453) zeigten sich neben der guten Durchführbarkeit und nachweislicher Verbesserung der körperlichen Aktivität Probleme bei der Zuteilung der Studienteilnehmer. Einige CAYAs führten bereits präventive Maßnahmen durch, wie beispielsweise eine adäquate sportliche Betätigung. Für diese CAYAs (ca. 20-30%) ist eine intensivierte Sportberatung demnach nicht sinnvoll, während in anderen Bereichen (z.B. Psychoonkologie) durchaus Bedarf bestand.

b. CARE for CAYA-Programm

Basierend auf diesen Erfahrungen ist demnach eine initiale umfassende Bedarfsanalyse gefolgt von einem bedarfsadaptierten Präventionsprogramm mit verschiedenen Interventionen sinnvoll, um möglichst effektiv und kosteneffizient eine individuell zugeschnittene, optimale und langfristige Versorgung zu gewährleisten.

Aus diesem Grund haben wir gemeinsam mit Experten der Versorgung von CAYAs in Deutschland (13 Kooperationspartner) und unter Einbeziehung und Beratung durch betroffene CAYAs das *CARE for CAYA-Programm* entwickelt.

Das Programm besteht an erster Stelle aus der Implementierung der Basisversorgung mit Bildung multiprofessioneller Teams unter Einbeziehung von Ärzten, Ernährungs- und Sportberatern sowie Psychoonkologen entsprechend der o.g. Empfehlungen an den einzelnen CAYA-Zentren [25, 43, 52, 83-85].

In einem weiteren Schritt soll die Effektivität einer zusätzlichen bedarfsadaptierten intensivierten Versorgung mit Interventionen bei CAYAs mit entsprechendem Bedarf in den Bereichen Ernährung, Sport und Psychoonkologie untersucht werden. Dies soll im Rahmen des initialen randomisierten Studienteils im Vergleich zur alleinigen Basisversorgung bei CAYAs mit hohem Interventionsbedarf erfolgen, um die Effektivität des Präventionsprogramms zu belegen.

Im Anschluss an den randomisierten Studienteil wird das Programm vorerst für den Zeitraum von 3 Jahren (Zeitraum der Finanzierung durch den Innovationsfond des Gemeinsamen Bundesausschuss, GBA) fortgesetzt zur weiteren Optimierung des Programmes und der einzelnen Interventionen. Der modulare Aufbau des Programms ermöglicht darüber hinaus die Anpassung/ Erweiterung der Module des *CARE for CAYA-Programms* auf zukünftige relevante Problembereiche durch Ergänzung entsprechender weiterer spezifischer Unterstützungsangebote oder Interventionen.

Langfristig sollen durch das *CARE for CAYA-Programm* Langzeitfolgen der Tumortherapie durch bedarfsadaptierte präventive Maßnahmen abgewendet, Folgeerkrankungen und psychosozialen Probleme reduziert und die Erwerbstätigkeit bzw. das Erlangen einer beruflichen Qualifikation erhalten bzw. wiederhergestellt werden.

3. Hypothesen

1.1 Primäre Hypothesen

Modulare Interventionen

- Die Teilnahme am *CARE for CAYA-Programm* mit den jeweiligen bedarfsadaptierten Interventionen führt zu einer Verbesserung des Lebensstils (Sport und/oder Ernährung) und/oder der psychosozialen Situation (Ablaufschema siehe Abbildung 1).

Bedarfsanalyse

- Die Verbesserung der Bedarfsanalyse im Rahmen des CARE for CAYA-Programms ermöglicht die Reduktion nicht-erfasster und damit nicht adressierbarer Bedürfnisse junger Krebsüberlebender.

a. Sekundäre Hypothesen

Das *CARE for CAYA-Programm*

- ist durchführbar,
- ist kosteneffizient,
- ermöglicht die adäquate und effektive bedarfsadaptierte Zuordnung mit Hilfe der jährlichen Erhebungen und verfügt über effektive Interventionen,
- verbessert das kardiovaskuläre Risikoprofil,
- verbessert die Lebensqualität.

Die Interventionsmodule verbessern im Bereich

- Sport die körperliche Aktivität, bauen Barrieren gegenüber körperlicher Aktivität ab und erhöhen die Leistungsfähigkeit,
- Ernährung das Ernährungsverhalten und die Körperzusammensetzung, das Körpergewicht, bauen Barrieren gegenüber gesunder Ernährung ab und verbessern die Geschmackswahrnehmung,
- Psychoonkologie die Patientenkompetenz, Depression, Ängste, Lebensqualität und Fatigue.

4. Endpunkte

1.1 Endpunkte des CARE for CAYA-Programms

Primäre Endpunkte:

Modulare Interventionen

- Rate der CAYAs mit hohem Interventionsbedarf nach 12 monatiger Intervention (Vergleich Interventions- und Kontrollgruppe im randomisierten Studienteil)

Bedarfsanalyse (Co-primärer Endpunkt)

- Rate der CAYAs mit noch nicht im Rahmen der Bedarfsanalyse charakterisierten Bedürfnisse

Sekundäre Endpunkte:

Durchführbarkeit

- Rate der Etablierbarkeit des Programmes im Konsortium (Vorhandensein räumlicher Gegebenheiten, Besetzung der Personalstellen, Implementierung des Programmablaufs)
- Monatliche Einschlussraten der Konsortialzentren
- Rate der vollständige ausgefüllten Fragebögen im Rahmen der Bedarfsanalyse
- Rate der vollständig erhobenen medizinischen und apparativen Diagnostik (z.B. BIA- oder ActiGraphen-Messung)
- Rate im Programm verbleibender CAYAs (mindestens zweimalige Bedarfsanalyse) in Relation zur Teilnahme an interventionellen Modulen
- Umsetzbarkeit der Adaptation der Bedarfsanalyse (Zeitrahmen zwischen Auswertung der Daten, Änderung des Protokolls, Einholung der Genehmigung durch die Ethikkommission und Implementierung der adaptierten Bedarfsanalyse)
- Drop-out-Raten (bezogen auf die Gesamtstudie und die einzelnen Interventionen)

Kosteneffizienz

- Kosteneffizienz (sekundäre Krankheitskosten, Gesundheitsökonomie und -management, Inanspruchnahme von Gesundheitsleistungen)

Allokation und Effektivität der modularen Interventionen

- Veränderungen der Bedürfnisse nach 12 Monaten in Relation zur Interventionsbedürftigkeit des initialen Bedarfs (Tabelle 1) und der Teilnahme an einem interventionellen Modul
- Prävalenz kardiovaskulärer Risikofaktoren (BMI, WHR, Blutzucker-/Fettstoffwechselstörungen, Arterieller Hypertonus)

- Lebensqualität und Fatigue

a. Endpunkte der Interventionsmodule

1) Sport und körperliche Aktivität

Primärer Endpunkt:

- körperliche Aktivität (Bewegungs- und Sportaktivität-Fragebogen, erweitert - BSA-Fragebogen, erweitert)[91] und Aktivitätsmessung

Sekundäre Endpunkte:

- regelmäßige körperliche Aktivitäts-Einheiten
- situative und sportbezogene Barrieren
- Lebensqualität und Fatigue (EORTC QLQ-C30 und FA12)[92]
- Phasenwinkel (BIA Messung)
- Ausdauerleistungsfähigkeit gemessen durch Spiroergometrie und Laktatdiagnostik (Subgruppe an einzelnen Zentren)

2) Ernährung

Primärer Endpunkt:

- Ernährungsverhalten (berechnet mit HEI-EPIC und Kurzscreening Ernährung)

Sekundäre Endpunkte:

- Phasenwinkel (BIA Messung)
- Verbesserung des MEDAS-FFQ
- ernährungsbezogene Barrieren
- Prävalenz von Untergewicht, Übergewicht oder Adipositas (BMI)
- Lebensqualität und Fatigue (EORTC QLQ-C30 und FA12)
- Prävalenz von subjektiver und objektiver Geschmacksbeeinträchtigung (Subgruppe an einzelnen Zentren)

3) Psychoonkologie

Primärer Endpunkt:

- Patientenkompetenz (CBI-B) [93]

Sekundäre Endpunkte:

- Selbstwirksamkeitserlebens (SWE) [94]
- Depressivität (PHQ-9) [95] und Distresserleben (NCCN DT) [96]
- Ängstlichkeit (GAD-7) [97] und Progredienzangst (PA-F-KF)[98]
- Lebensqualität und Fatigue (EORTC QLQ-C30 und FA12)

5. Auswahlkriterien

- Patienten mit einer hämato-onkologischen Erkrankung im Kindes-, Jugend- und jungen Erwachsenen Alter (CAYAs), die sich nach abgeschlossener Therapie in der Nachsorge befinden und aktuell tumorfrei sind
- Patienten mit einem Alter ≥ 15 Jahre und ≤ 39 Jahre
- schriftliche Einverständniserklärung (bei CAYAs ≤ 18 schriftliches Einverständnis durch alle Erziehungsberechtigten)

- keine schwerwiegenden, behandlungsbedürftigen Erkrankungen in den im Rahmen des Präventionsprogrammes adressierten Bereichen (z.B. manifeste psychische Erkrankung)

6. Studiendesign

1.1 Organisatorische Struktur des CARE for CAYA-Programms

Das Programm wird deutschlandweit in regionalen Zentren (15 Kooperationszentren) implementiert, in denen bereits Strukturen mit Sprechstunden zur medizinischen Tumornachsorge bei CAYAs bestehen und damit eine besondere Expertise in der Betreuung dieser speziellen Patientengruppe gewährleistet ist. Dabei handelt es sich ausschließlich um Kliniken mit pädiatrisch-onkologischen Zentren, interdisziplinären Sprechstunden und entsprechender Expertise im Bereich der Survivorship-/CAYA-Nachsorge sowie Forschungsaktivität und Mitarbeit in bundesweiten Gremien und Arbeitsgruppen zur Versorgung von CAYAs oder von Langzeitnebenwirkungen in den Fachgesellschaften (Gesellschaft für Pädiatrische Onkologie und Hämatologie – GPOH und/oder Deutsche Gesellschaft für Hämatologie und Onkologie – DGHO) (siehe angegebene Konsortialzentren). Die beteiligten Zentren leiten dabei klinische Studiengruppen oder Register u.a. „Cooperative Osteosarkom Studiengruppe – COSS“ in Stuttgart, „Late Effects Surveillance System – LESS“ in Lübeck, „Europäische Studie zur Behandlung von intrakranialen Keimzelltumoren – SIOP CNS GCT“ in Bonn zur Therapie und Nachsorge von CAYAs und verfügen demnach über eine herausragende Expertise auf diesem Gebiet.

Das *CARE for CAYA-Programm* soll in an folgenden Konsortialzentren umgesetzt werden:

- Bonn
- Erlangen
- Essen
- Freiburg
- Hamburg
- Hannover
- Jena
- Lübeck
- Magdeburg
- Mainz
- Münster
- Rostock
- Stuttgart
- Würzburg

Die Konsortialführung in Hamburg verfügt im Rahmen des 2007 gegründeten Universitären Cancer Centers Hamburg (UCCH) über etablierte interdisziplinäre Strukturen zur Versorgung und wissenschaftlichen Begleitforschung in dieser Patientengruppe, insbesondere im Bereich Lebensstilverbesserung. So verfügt das seit mehr als 5 Jahren bestehende *Leben nach Krebs-Programm* über etablierte gemeinsame Sprechstunden und regelmäßige interdisziplinäre und multiprofessionelle Fallkonferenzen, sowie ein angeschlossenes Begleitforschungsprogramm mit Register- und Interventionsstudien für CAYAs [46, 82, 90].

Der klinische Teil des *CARE for CAYA-Programms* wird an den jeweiligen Zentren vor Ort durch qualifizierte Fachkräfte aus den jeweiligen Bereichen (Sport, Ernährung, Psychoonkologie) gewährleistet.

Die zentrale Datenerfassung der erhobenen Daten aus den einzelnen Standorten erfolgt am Interdisziplinären Centrum für Biobanking-Lübeck (ICB-L) an der Universität zu Lübeck (Konsortialpartner). Das ICB-L verfügt über eine langjährige Expertise in der Erfassung und Verarbeitung von klinischen Daten und hat bereits eine bestehende Datenbank zur Erfassung medizinischer Daten von Langzeitüberlebenden Krebspatienten (Patientenkohorte von Prof. Langer, Lübeck). Im Rahmen des Konsortiums wird die zentrale Datenerfassung im Rahmen von einer schriftlichen Vereinbarung zwischen dem ICB-L und den Konsortialzentren geregelt.

Die Auswertung der Daten erfolgt bei der Konsortialführung in Hamburg. Die Auswertungen und die sich daraus ergebenden, eventuellen Anpassungen des *CARE for CAYA-Programms* werden gemeinsam mit den Kooperationspartnern im Rahmen der regelmäßigen Konsortialtreffen (Präsenz und Telefonkonferenzen) diskutiert und entschieden. Für die einzelnen Module wurden entsprechend der eingebrachten Expertise Verantwortliche bzw. eine wissenschaftliche Leitung aus dem Konsortium benannt.

Das dem Programm zugrundeliegende Studienprotokoll und die Einwilligungserklärungen werden den jeweiligen lokalen Ethikkommissionen zur Begutachtung vorgelegt. Die Ethikkommission der Ärztekammer Hamburg wird dabei als federführende Ethikkommission fungieren.

a. Ablauf und Methodik des CARE for CAYA-Programms

1.1.1 Evaluation

Evaluiert werden sollen die bedarfsadaptierten Interventionen durch die randomisiert-kontrollierte Studie (siehe Abbildung 1) in der Gruppe der CAYAs mit hohem Bedarf.

Diese werden anhand der in der Bedarfsanalyse erhobenen Kriterien identifiziert (siehe Tabelle 1). Die Rate der CAYAs, die bei Beginn der Teilnahme im *CARE for CAYA-Programm* einen hohen Bedarf hinsichtlich der aktuellen, interventionellen Module (Sport, Ernährung und Psychoonkologie) haben, wird aufgrund eigener Registerdaten bei 60% erwartet. In der Gruppe der CAYAs mit hohem Bedarf soll die Effektivität der bedarfsadaptierten Interventionen durch eine Verminderung der Rate (an CAYAs mit hohem Bedarf) nachgewiesen werden (primäres Zielkriterium).

Alle 12 Monate bekommen alle CAYAs einen Tablet-basierten Basis- und Screeningfragebogen der sich aus verschiedenen evaluierten und validierten Fragebögen zusammensetzt. Dieses Screening besteht aus:

- NCCN Distress Thermometer (DT) [96]
- EORTC QLQ-C30 [99]
- Freiburger Ernährungsprotokoll (über 3 Tage) [100]
- Kurzscreening Ernährung
- Abfrage zu körperlicher Aktivität mit Borg-Skala [101] (Screeningfragen Modul Sport)
- Bewegungs- und Sportaktivität (BSA)-Fragebogen [91], erweitert mit Borg-Skala [101]
- Thermometer zur Abfrage körperlicher Aktivität
- Ultrakurzform des Gesundheitsfragebogens für Patienten (PHQ-4) [102]
- Abfrage von nicht adressiertem Unterstützungsbedarf
- Zufriedenheits-Fragebogen ZUF-8, modifiziert [103]
- Abfrage des Bedarfs
- Gesundheitsbezogene Lebensqualität (EQ5D-5L) [104]
- Auszug aus Fragebogen zu Leistungsvermögen in Schule, Ausbildung und bei der Arbeit von Jugendlichen und jungen Erwachsenen [105]

- Auszug aus Fragebogen zu Arbeitsausfall bei Erwachsenen [106]
- Kurzfragebogen Inanspruchnahme medizinischer Leistungen [107] [108]
- SCNS-TF-9 [109]

Auf Basis dieser Fragebögen wird ein hoher bzw. niedriger Bedarf erhoben, also die Zuordnung in die Module mit hohem oder niedrigem Bedarf durchgeführt (siehe Tabelle 1).

Zudem sind ein Teil der Fragen als sogenannte „Red Flags“ (Indexfragen) mit weiteren Fragebögen hinterlegt, die nur an die Patienten weitergereicht werden, wenn sich durch die Beantwortung der vorherigen Fragen ein Problem vermuten lässt. Somit sind beschwerdefreie oder -arme Patienten nicht durch das Ausfüllen von zusätzlichen Fragebögen zeitlich eingebunden. Durch die zusätzlichen Fragebögen bei den belasteten Patienten soll eine weitere Differenzierung der Symptomatik ermöglicht werden (bspw. Depression versus Fatigue).

Die Feststellung eines Interventionsbedarfs erfolgt im Modul Psychoonkologie auf der Basis von NCCN Distress Thermometer und PHQ-4. Wird beim NCCN Distress Thermometer ein Wert ≥ 5 oder wird PHQ-4 ein Wert ≥ 6 Punkte erreicht, dann wird der Patient gebeten, zusätzlich folgende psychoonkologischen Fragebögen auszufüllen:

- Depressionsmodul des Gesundheitsfragebogens für Patienten (PHQ-9) [95]
- Angstmodul des Gesundheitsfragebogens für Patienten (GAD-7) [97]
- Fatigue-Modul des EORTC (EORTC QLQ-FA12) [92]
- Allgemeine Selbstwirksamkeitserwartung (SWE) [94]
- Cancer Behavior Inventory – Brief Version (CBI-B) [93]
- Progredienzangstfragebogen – Kurzversion (PA-F-KF) [98]

Bei Feststellung eines Interventionsbedarfs laut Basis- und Screeningfragebogen im Rahmen des Moduls Sport sind zusätzlich folgende Fragebögen auszufüllen:

- Fragen zur Einstellung zu körperlicher Aktivität (TTM) [110]
- Skalen Sportbezogene situative Barrieren und Sportbezogenes Barrieremanagement [111]
- Bewegungstagebuch mit Borg-Skala [101]
- Borg-Skala [101] (im Rahmen des Bewegungstagebuchs und der Spiroergometrie , zentrumsspezifische erweiternde Untersuchung)

Bei Feststellung eines Interventionsbedarfs laut Basis- und Screeningfragebogen im Rahmen des Moduls Ernährung sind zusätzlich folgende Fragebögen auszufüllen:

- TTM - Stufenalgorithmus zur Ernährung [112]
- Entscheidungsbalance Kurzform [112]
- Fragebogen zu mediterranen Diät MEDAS-FFQ [113]
- Anamnesebogen zur Erhebung des Geruchs- und Geschmacksempfindens, nach Prof. Dr. Hummel, Universität Dresden (im Rahmen des Geschmackstests, zentrumsspezifische erweiternde Untersuchung)

Neben der Fragebogenerhebung werden ebenfalls jährlich medizinische Parameter zur Einschätzung des Interventionsbedarfs erhoben:

- Erfassung spezifischer demographischer und krankheitsbezogener Parameter
    - nur einmalig bei Studieneinschluss: Alter, Geschlecht, Zeitpunkt der Erstdiagnose, Diagnose, Therapie, familiäre Krebsvorkommen,

  - im Rahmen der jährlichen Erhebungen: bekannte und neu-aufgetretene Nebenerkrankungen, Nikotin-, Drogen- und Alkoholabusus, aktuelle Medikation, Inanspruchnahme medizinischer Leistungen und Einrichtungen
- Erfassung von Blutdruck (systolisch, diastolisch), Größe, Gewicht und BMI, Waist-Hip-Ratio, (WHR), Herzfrequenz
- Weitere Kriterien des metabolischen Syndroms (arterielle Hypertonie, Gesamt-Cholesterin, LDL-Cholesterin, HDL-Cholesterin, Triglyzeride, Blutglucose, HbA1c)

Eine Evaluation der Bedarfsanalyse bzgl. der Rate der CAYAs mit noch nicht im Rahmen der Bedarfsanalyse charakterisierten Bedürfnisse, Durchführbarkeit und der adäquaten Allokation in die jeweiligen Interventionen (siehe Endpunkte) ist nach der 300. Bedarfsanalyse geplant. Während der mit initialem Fragebogen fortgesetzter Bedarfsanalyse erfolgt die Evaluation, Anpassung anhand der erhobenen Ergebnisse und letztlich Implementierung nach Erhalt des Ethikvotums. Geplant ist diese Anpassung in einem zeitlichen Rahmen von ca. 4-5 Monaten zu erreichen. In diesem Zeitraum werden planmäßig weitere 250 Bedarfsanalysen mit dem initialen Fragebogen erhoben. Ab dem 562. CAYA wird dann die adaptierte Bedarfsanalyse zum Einsatz kommen und bis zum Ende des Programmes am 31.07.2020 bzw. zum Einschluss des 1124. CAYAs angewendet. In der finalen Evaluation sollen dann die beiden Gruppen (initiale Bedarfsanalyse 1.-561. CAYA vs. adaptierte Bedarfsanalyse 562.- 1124. CAYA) vergleichend evaluiert werden (Co-primärer Endpunkt).

Um einen gegebenenfalls kurzfristigeren Interventionseffekt nachweisen zu können, werden in der Interventionsgruppe nach 16 +/- 4 Wochen (abhängig von individuell vereinbarten Terminen mit mind. 2 Wochen Abstand zur letzten psychoonkologischen Intervention und frühestens nach der Woche 12 Beratung in der Sport- oder Ernährungsinterventionsgruppe) und in der Kontrollgruppe ebenfalls nach 16 +/- 4 Wochen erneut folgende Fragebögen erhoben:

- NCCN Distress Thermometer (DT) [96]
- EORTC QLQ-C30 [99]
- Freiburger Ernährungsprotokoll (über 3 Tage) [100]
- Kurzscreening Ernährung
- Abfrage zu körperlicher Aktivität mit Borg-Skala [101] (Screeningfragen Modul Sport)
- Bewegungs- und Sportaktivität (BSA)-Fragebogen [91], erweitert mit Borg-Skala [101]
- Thermometer zur Abfrage körperlicher Aktivität
- Ultrakurzform des Gesundheitsfragebogens für Patienten (PHQ-4) [102]
- Abfrage von nicht adressiertem Unterstützungsbedarf
- Zufriedenheits-Fragebogen ZUF-8, modifiziert [103]
- Abfrage des Bedarfs
- Gesundheitsbezogene Lebensqualität (EQ5D-5L) [104]
- Auszug aus Fragebogen zu Leistungsvermögen in Schule, Ausbildung und bei der Arbeit von Jugendlichen und jungen Erwachsenen [105]
- Auszug aus Fragebogen zu Arbeitsausfall bei Erwachsenen [106]
- Kurzfragebogen Inanspruchnahme medizinischer Leistungen [107] [108]
- SCNS-TF-9 [109]

Zusätzlich werden bedarfsspezifisch die folgenden Fragebögen ausgefüllt

- Depressionsmodul des Gesundheitsfragebogens für Patienten (PHQ-9) [95]
- Angstmodul des Gesundheitsfragebogens für Patienten (GAD-7) [97]
- Fatigue-Modul des EORTC (EORTC QLQ-FA12) [92]

- Allgemeine Selbstwirksamkeitserwartung (SWE) [94]
- Cancer Behavior Inventory – Brief Version (CBI-B) [93]
- Progredienzangstfragebogen – Kurzversion (PA-F-KF) [98]
- Fragen zur Einstellung zu körperlicher Aktivität (TTM) [110]
- Skalen Sportbezogene situative Barrieren und Sportbezogenes Barrieremanagement [111]
- Bewegungstagebuch
- Borg-Skala [101] (im Rahmen der Spiroergometrie, zentrumsspezifische erweiternde Untersuchung)
- TTM - Stufenalgorithmus zur Ernährung [112]
- Entscheidungsbalance Kurzform [112]
- Fragebogen zu mediterranen Diät MEDAS-FFQ [113]
- Anamnesebogen zur Erhebung des Geruchs- und Geschmacksempfindens (nach Prof. Dr. Hummel, Universität Dresden) (im Rahmen des Geschmackstests, zentrumsspezifische erweiternde Untersuchung)

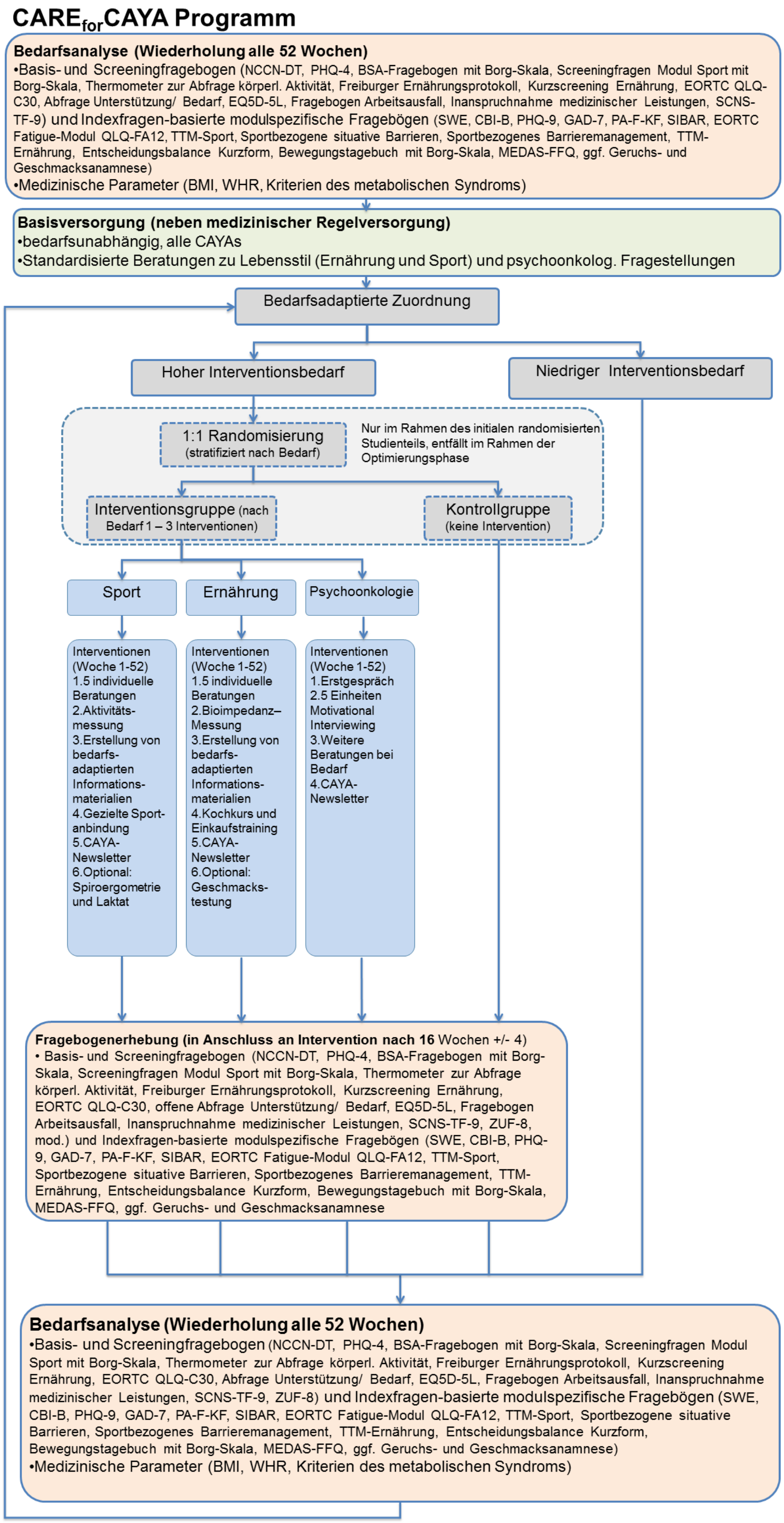

Abbildung 1 Design des CARE for CAYA-Programms

i. Basisversorgung im CARE for CAYA-Programm

Allen CAYA-Patienten der regulären Tumornachsorge-Sprechstunden wird die Teilnahme am *CARE for CAYA-Programm* empfohlen. Nach Einschluss und nach erfolgter Bedarfsanalyse (Vorgang der Bedarfsanalyse siehe Punkt 7.2.1) erhalten *alle* CAYAs *unabhängig vom individuellen Bedarf* eine Basisversorgung. Die Basisversorgung umfasst neben der medizinisch notwendigen Nachsorge das Angebot einer Beratung in den Bereichen Ernährung, Sport und Psychoonkologie unabhängig des im Basis- und Screeningfragebogen und des anhand medizinischer Parameter erhobenen Bedarfs. Hierfür wird der Kontakt zu dem jeweiligen Berater hergestellt. Dabei geht es um eine einmalige Beratung und Aufklärung, bei der allgemeine schriftliche Informationen zum Thema Ernährung und Sport zur Verfügung gestellt werden, jedoch keine fortlaufenden oder regelmäßigen Informationen oder weitere Interventionen stattfinden.

CAYA-Patienten mit Therapieindikation beispielsweise bei manifester psychischer Erkrankung werden nach Identifikation durch den Arzt und/oder den Psychoonkologen etc. der jeweiligen notwendigen Therapie zugeführt.

Im Rahmen der Basisberatung sollen Bedürfnisse geklärt werden, die nicht in den standardisierten Fragebögen erfasst werden. Ebenso gibt es für CAYAs das niedrigschwellige Angebot, Entlastungsgespräche mit einem Psychoonkologen wahrzunehmen.

Um eine standardisierte Basisversorgung gewährleisten zu können, sind für alle Zentren Manuale für die Bereich Sport, Ernährung und Psychoonkologischer Erstkontakt erstellt.

Die psychoonkologische Basisversorgung erfolgt im Rahmen klientenzentrierter supportiver Gespräche.

Für die CAYA-Patienten mit hohem Interventionsbedarf werden ebenfalls standardisierte Beratungsleitfaden und Anamnesefragebogen für die Module Sport und Ernährung erstellt, die dann angewandt werden, wenn ein Einschluss in die entsprechenden Module erfolgt. Die psychoonkologische Versorgung in dem intensiv betreuten Modul erfolgt nach den Vorgaben des *Motivational Interviewing*.

ii. Bedarfsadaptierte Versorgung: Allokation in eine Gruppe mit hohem Interventionsbedarf und niedrigem Interventionsbedarf

Entsprechend der Bedarfsanalyse erfolgt eine Zuordnung der CAYAs in eine Gruppe mit hohem Interventionsbedarf und eine Gruppe mit niedrigem Interventionsbedarf. CAYAs mit einem hohen Interventionsbedarf werden nach Randomisierung den Interventionen des entsprechenden Moduls (siehe Abbildung 1) zugewiesen. Ein CAYA kann in mehreren Interventions-Modulen einen erhöhten Bedarf aufweisen und entsprechend mehrere Interventionen benötigen.

Ein hoher Bedarf wird definiert als nicht adäquates Ernährungsverhalten und/oder körperliche Aktivität und/oder relevante psychosoziale Beeinträchtigungen. Entsprechende modulspezifische Stratifikationskriterien sind in Tabelle 1 zusammengestellt.

Modul	Hoher Interventionsbedarf (mindestens ein Kriterium zutreffend)
**Sport und körperliche Aktivität**	• < 150 Minuten/Woche moderate körperliche Aktivität und/oder < 75 Minuten intensive körperliche Aktivität<br>• < 3 Tage körperliche Aktivität die Woche (moderat und/oder intensiv), davon < 2 Tage intensive körperliche Aktivität die Woche<br>• Nachweis von Kriterien des metabolischen Syndroms
**Ernährung**	• ~~≤ 40 HEI-EPIC-Punkte~~ (bis Version 07)

	• Kurzscreening Ernährung; Score ≤ 29 Punkte (ab Version 7)<br>• BMI < 18,5 oder ≥ 30<br>• WHR bei Frauen > 0,85, WHR bei Männern > 1,0<br>• Anamnestisch gastrointestinale Beschwerden (z.B. Durchfälle, Unverträglichkeiten)<br>• Nachweis von Kriterien des metabolischen Syndroms
**Psychoonkologie**	• ≥ 6 Punkte im PHQ- 4<br>• ≥ 5 Punkte NCCN-DT

Tabelle 1 Zuordnungskriterien für hohen Interventionsbedarf

iii. Randomisierung der CAYAs mit hohem Interventionsbedarf in Interventionsgruppe und Kontrollgruppe (nur in der initialen Studienphase des *CARE for CAYA-Programms*)

Alle CAYAs werden im Rahmen des *CARE for CAYA-Programms* mit Hilfe der modularen Zuordnungskriterien in die Gruppen „hoher Bedarf“ (Basisversorgung und Intervention/-en) und „niedriger Bedarf“ (alleinige Basisversorgung) eingeteilt (siehe Tabelle 1). Nach dem jeweiligen Bedarf erfolgt in der initialen Studienphase die Randomisation (1:1) in eine Interventionsgruppe oder Kontrollgruppe. Die Randomisation wird zentral für alle Zentren durchgeführt und nach Zentrum stratifiziert. Die CAYAs, welche in die Interventionsgruppe randomisiert werden, erhalten die intensivierten modularen Interventionen. CAYAs, die im Rahmen der klinischen Studie in der Kontrollgruppe betreut werden und nach 12 Monaten in der erneuten Bedarfsanalyse nach Zuordnungskriterien (siehe Tabelle 1) weiterhin einen erhöhten Bedarf aufweisen, erhalten dann ihre intensivierten modularen Interventionen. Die Randomisation im Rahmen der Studie erfolgt dementsprechend einmalig. Die Stratifikation wird nach einem Jahr wiederholt.

iv. *CARE for CAYA* Module und Interventionen

Das *CARE for CAYA-Programm* beinhaltet Interventionsmodule und Evaluationsmodule, sowie im Rahmen der initialen randomisierten Phase eine Kontrollgruppe (bzw. Warteliste).

Für die Betreuung der Patienten in allen Gruppen werden standardisierte Vorgehensweisen in schriftlicher Form als Manuale vorgegeben, um die Qualität der Interventionen zu gewährleisten.

Sollten sich die Interventionen hinsichtlich den unter Punkt 8.2. definierten Veränderungen der Endpunkte als nicht effektiv erweisen oder ist die Zuordnung zu den Interventionen oder der Fragebogen nicht adäquat, dann sind entsprechende Anpassungen des Programms geplant, die dann in Form eines Amendments erneut der Ethikkommission vorgelegt werden.

<u>**Interventionsmodule**</u>

1) Sport und körperliche Aktivität

Im Modul Sport besteht die Intervention aus 5 individuellen Beratungen mit weiterführenden Unterstützungsangeboten sowie regelmäßiger Zusendung von schriftlichen Informationen per Post.

Der Sporttherapeut erstellt dabei einen individuellen Bewegungsplan unter Berücksichtigung persönlicher evtl. krankheitsbedingter Bewegungseinschränkungen, vermittelt Sportangebote und erarbeitet gemeinsam mit dem Patienten eine Zielvorstellung.

Beratungsgespräche (Woche 0, 6, 12, 18, 24 +/- 4 Wochen) á 60 Minuten

- Anamnese: Erfragung der aktuellen körperlichen und sportlichen Aktivität: Bewegung im Alltag, sitzende oder stehende Tätigkeit, ggf. körperliche Belastung bei der Arbeit, Arbeitsweg (zu Fuß, Fahrrad, öffentliche Verkehrsmittel, Auto), aktuell ausgeübter Sport, Intensitäten, persönliche „Sportbiografie“, Schulsport, Betriebssport etc.
- Individuelle, bedarfsadaptierte Beratung zu Sport und körperlicher Aktivität
- Erstellung eines Bewegungsplans unter Berücksichtigung persönlicher (evtl. krankheitsbedingter) Bewegungseinschränkungen sowie Erarbeitung einer Zielvorstellung [114]
- Erörterung der sportbezogenen situativen Barrieren und Entwicklung von Gegenstrategien
- Erläuterungen und Anlegen des Aktivitätsmessers (Akzelerometrie) mit Messungen in Woche 0, 16 und 52 +/- 4 Wochen
- Durchführung einer Bioimpedanzanalyse (BIA)-Messung (Messung in Woche 0, 16 und 52 +/- 4 Wochen)
- Bereitstellung von allgemeinen Informationsmaterialien zum Thema Sport und körperliche Aktivität, u.a. den blauen Ratgeber „Bewegung und Sport bei Krebs“
- Unterstützung der gemeinsamen Aktivitäten im Rahmen des sozialen Umfelds (Freunde, Familie)
- Im Rahmen der weiteren Beratungsgespräche werden die Ergebnisse der erhobenen objektiven Befunde (Blutdruckmessung, ggf. 24-Stunden-Blutdruckmessung, BMI, WHR, Akzelerometrie) besprochen und Verbesserungsmöglichkeiten aufgezeigt, sowie die Erreichung vereinbarter Ziele überprüft und ggf. angepasst
- Fragen zur Einstellung zu körperlicher Aktivität (TTM) in Woche 0, 16 und 52 +/- 4 Wochen
- Skalen „Sportbezogene situative Barrieren“ und „Sportbezogenes Barrieremanagement“ [111] in Woche 0, 16 und 52 +/- 4 Wochen
- Zentrumsspezifische erweiternde Untersuchung in Hamburg und optional in weiteren Zentren: Spiroergometrie und Laktatdiagnostik in Woche 0, 16 und 52 +/- 4 Wochen

2) Ernährung

Im Modul Ernährung besteht die Intervention aus 5 individuellen Beratungen mit weiterführenden Unterstützungsangeboten sowie regelmäßiger Zusendung von angepassten schriftlichen Informationen per Post. Vom Ernährungstherapeuten werden eine individuelle Beratung mit Erstellung eines Ernährungsplans, Besprechung persönlicher Probleme und Aufklärung über Lebensmittel und ihrer Zusammensetzung durchgeführt.

Zusätzlich werden in der Gruppe ein Einkaufstraining und ein Kochkurs durchgeführt, um grundsätzliche Aspekte einer ausgewogenen Ernährung zu vermitteln.

Beratungsgespräche (Woche 0, 6, 12, 18, 24 +/- 4 Wochen) á 60 Minuten

- Aushändigung von zusätzlichen Informationsmaterialien zum Thema gesunde und ausgewogene Ernährung nach einer Krebserkrankung, u.a. den blauen Ratgeber „Ernährung bei Krebs“ und „10 Regeln der DGE“.

- Individuelle Ernährungsberatung über eine gesunde und ausgewogene Ernährung im Rahmen der Krebsprävention, Durchführung einer ausführlichen Ernährungsanamnese und einer persönlichen Auswertung der Ernährungsprotokolle, Anfertigung eines individuellen Ernährungsplans unter Berücksichtigung der gemeinsamen Zielvereinbarung
- Erörterung der ernährungsbezogenen Barrieren und Entwicklung von Gegenstrategien
- Durchführung einer Bioimpedanzanalyse (BIA)-Messung (Messung in Woche 0, 16 und 52 +/- 4 Wochen) und Geschmackstest (Messung in Woche 0 und 52 +/- 4 Wochen)
- Anamnese: Anthropometrie, Gewichtsverlauf, Blutdruck (systolisch, diastolisch), Medikamenteneinnahme, Essverhalten/Essgewohnheiten, Nikotin- und Alkoholkonsum, Stuhlgang, Allergien/Unverträglichkeiten, Vitalität, Motivation, Ziele/Wünsche/Fragen (Woche 0, 16 und 52 +/- 4 Wochen)
- Im Rahmen der weiteren Beratungsgespräche werden die Ergebnisse der erhobenen objektiven Befunde (Blutdruckmessung, ggf. 24-Stunden-Blutdruckmessung, BMI, WHR, Bioimpedanzanalyse, Geschmackstest) besprochen und Verbesserungsmöglichkeiten aufgezeigt, sowie die Erreichung vereinbarter Ziele überprüft und ggf. angepasst
- TTM - Stufenalgorithmus zur Ernährung in Woche 0, 16 und 52 +/- 4 Wochen
- „Entscheidungsbalance Kurzform" in Woche 0, 16 und 52 +/- 4 Wochen
- Zentrumsspezifische erweiternde Untersuchung in Hamburg und optional in weiteren Zentren: Geruchs- und Geschmacksanamnese sowie Geschmackstest in Woche 0, 16 und 52 +/- 4 Wochen

Einkaufstraining und Kochkurs (Woche 8 - 20+/- 4 Wochen)

Ab Woche 12 bis Woche 36 erhalten alle Patienten der Interventionsgruppe „Ernährung" einmalig einen Kochkurs und ein Einkaufstraining. Der Kochkurs findet in kleinen Gruppen von max. 8 CAYAs unter Anleitung des Ernährungsberaters statt. In einem Zeitraum von 2-3 Stunden sollen die CAYAs lernen, gesunde und ausgewogene Speisen zuzubereiten. Zusätzlich wird ein Einkaufstraining angeboten, welches ebenfalls in kleinen Gruppen (max. 5 Personen) in einem Zeitraum von 45-60 Minuten in einem beliebigen Supermarkt durchgeführt wird. Hierbei sollen die CAYAs grundsätzliches zur Zusammensetzung von verarbeiteten Lebensmitteln erfahren, die Nährstoffangaben auf Lebensmittelverpackungen zu deuten lernen und gesunde Alternativen dazu kennenlernen.

Regelmäßige Bereitstellung individueller Informationen zu körperlicher Aktivität, Ernährung und psychosozialen Themen – CAYA Newsletter (alle 6-8 Wochen):

- Zusammenfassung der Sport- und Ernährungsberatung sowie konkrete Empfehlungen zur Verbesserung
- Information über die individuellen Ergebnisse der Aktivitätsmessung, BIA Messung, HEI-EPIC und Kurzscreening Ernährung.
- gezielte Vermittlung von Sportangeboten: Angebote in der Nähe des Wohnortes oder der der Arbeit (gemeinnützige und gewerbliche Anbieter, spezielle Angebote für CAYAs z.B. Segelrebellen, Rehasport-Gruppen, Physiotherapie, Angebote der Krankenkassen: Gesundheitskurse, Leistungsdiagnostik

- gezielte Informationen zu sport- und bewegungsrelevanten Themen, u.a. Kraft- und Ausdauertraining, Bewegung im Alltag, Sporternährung, Sport in der Prävention
- gezielte Informationen für eine gesunde und ausgewogene Ernährung, z.B. hochwertige Kohlenhydrate und Ballaststoffe, die richtige Fettauswahl, Hinweis auf verstecktes Salz und gesunde Alternativen, einfache und gesunde Kochrezepte
- gezielte Informationen zu psychologischen Themen, u.a. Krankheitsverarbeitung, Stressmanagement, Entspannungstrainings, Selbstfürsorge, seelische Gesundheit

3) Psychoonkologie (Interventionsmodul)

Im psychosozialen Modul werden den Patienten mehrere Einzelgespräche mit Hilfe des *Motivational Interviewing*-Verfahrens angeboten. *Motivational Interviewing* ist ein sowohl patientenzentrierter als auch geleiteter Ansatz der therapeutischen Gesprächsführung mit dem Ziel, die Eigenmotivation zu erhöhen und durch Verhaltensveränderung ein persönlich relevantes Ziel zu erreichen.

Psychoonkologische Einzelintervention *Motivational Interviewing* (5 Termine nach Absprache)

Die Einzelintervention erfolgt unter Anwendung von *Motivational Interviewing* mit dem primären Ziel der Erhöhung der Patientenkompetenz und Selbstwirksamkeit in Auseinandersetzung mit den subjektiven Anforderungen. In einem Erstgespräch erfolgt neben der Anamneseerstellung die Auswahl eines persönlich relevanten Veränderungsziels des Patienten, welche Grundlage für die sich daran anschließenden fünf Sitzungen à 50 Minuten des *Motivational Interviewing* sein wird. Die Einzelintervention wird von DKG-zertifizierten (z.B: WPO Curriculum) Psychoonkologen mit Weiterbildung in *Motivational Interviewing* angeboten.

<u>Evaluationsmodule</u>

Neben den Interventions-Modulen werden auch Evaluations-Module eingesetzt, um den eventuell bestehenden Bedarf in anderen Problemstellungen bei den Patienten zu definieren und bei ggf. erhöhtem Bedarf perspektivisch neue Interventionen planen zu können. Die Evaluations-Module werden im Rahmen der Indexfragen und weiterführender Fragebögen eingesetzt. Es werden Bedarfserhebungen in den Bereichen chronisches Erschöpfungssyndrom – Modul Fatigue und kognitive Dysfunktion – Modul Neurokognition durchgeführt. Die Aufnahme einer Intervention bei bestehendem Bedarf kann aufgrund des modularen Aufbaus des *CARE for CAYA-Programms* jederzeit erfolgen, um das laufende Gesamtprogramm weiter zu ergänzen.

b. Zeitliche Abläufe des CARE for CAYA-Programms

CAYA-Patienten können jederzeit in das *CARE for CAYA-Programm* aufgenommen werden.

Im jährlichen Abstand wird im Rahmen der regulär erfolgenden Tumornachsorgevisiten die Bedarfsanalyse wiederholt, um die Bedarfsraten zu erheben und zu analysieren, aber auch den CAYAs entsprechend dem jeweiligen aktuellen Bedarf ggf. erneut Interventionen in den entsprechenden Modulen anzubieten.

Bei CAYAs mit erhöhtem Bedarf findet die Bedarfsanalyse weiter im jährlichen Intervall statt.

Besteht kein erhöhter Bedarf, wird die Bedarfsanalyse erneut im Folgejahr und im weiteren alle zwei Jahre wiederholt.

Die folgende Tabelle gibt eine Übersicht über den zeitlichen Ablauf und Aufwand für die Interventionen innerhalb der einzelnen Module, die nach der Bedarfsanalyse ermittelt wurden. Je nach Bedarfsanalyse kann ein Patient an 1-3 Modulen teilnehmen.

Modul	Zeitlicher Ablauf / Aufwand	Intervention / Untersuchung
**Sport und körperliche Aktivität**	Woche 0	• Baseline Assessment
	Insgesamt 5 Beratungen á 60 Minuten in Woche 0, 6, 12, 18, 24 +/-4 Wochen	• Individuelle Beratung zu Sport und körperlicher Aktivität
	Messungen in Woche 0, 16 und 52 +/- 4 Wochen	• Erläuterungen und Anlegen des Aktivitätsmessers (Akzelerometrie), der eine Woche lang getragen wird<br>• Rücksendung des Aktivitätsmessers per Post<br>• Durchführung der Bioimpedanzanalyse (BIA)–Messung
	Messung in Woche 0, 16 und 52 +/- 4 Wochen	• Spiroergometrie (zentrumsspezifische erweiternde Untersuchung)
	Innerhalb von Woche 0-52 alle 6 Wochen +/- 4 Wochen	• Newsletter mit Informationen zu Ernährung und körperlicher Aktivität
**Ernährung**	Woche 0	• Baseline Assessment
	Insgesamt 5 Beratungen á 60 Minuten in Woche 0, 6, 12, 18, 24 +/-4 Wochen	• Individuelle Ernährungsberatung
	Messung in Woche 0, 16 und 52 +/-4 Wochen	• Durchführung der Bioimpedanzanalyse (BIA)–Messung
	Messung in Woche 0, 16 und 52+/- 4 Wochen	• Durchführung der Geruchs- und Geschmacksanamnese sowie Geschmackstest (zentrumsspezifische erweiternde Untersuchung)
	Zwischen Woche 8 und 20 +/-4 Wochen einmalig nach Absprache, Dauer: 45-60 Minuten (in Gruppen von max. 5 Teilnehmer)	• Einkaufstraining
	Zwischen Woche 8 und 20–+/-4 Wochen einmalig nach Absprache, Dauer: 2-3 Stunden (in Gruppen von max. 8 Teilnehmer)	• Kochkurs
	Innerhalb von Woche 0-52 alle 6 Wochen +/- 4 Wochen	• Newsletter mit Informationen zu Ernährung und körperlicher Aktivität
**Psychoonkologie**	Intervention: 5 Termine à 50 Minuten nach Absprache (über einen Zeitraum von 2-4 Monaten).	• Einzelgespräche: Motivational Interviewing

	Innerhalb von Woche 0-52 alle 6 Wochen +/- 4 Wochen	• Newsletter mit Informationen zu Krankheitsverarbeitung, Selbstfürsorge und seelischer Gesundheit

Tabelle 2 Übersicht der Interventionen

7. Statistik

1.1 Auswertung

Die Evaluation des CARE for CAYA Programms erfolgt in verschiedenen Kohorten.

**Kohorten**	**Auswertungszeitraum**
Alle CAYAs mit auswertbarer erstmaliger Bedarfsanalyse (Gesamtkohorte, n=1124)	1.12.2020-31.03.2021 bzw. 31.07.2021 Zwischenauswertung nach 300. Patienten (ca. 1.12.2018-31.01.2019)
CAYAs mit mindestens zwei verfügbaren Bedarfsanalysen im Abstand von 12 Monaten (n=600)	1.12.2020-31.03.2021 bzw. 31.07.2021
Randomisierte CAYAs (n=320)	12 Monate nach Einschluss des letzten randomisierten Patienten ca. 3. Quartal 2020

Tabelle 3 Kohorten und Auswertungszeitraum

Für den primären Endpunkt Bedarfsanalyse und die sekundären Endpunkte zur Durchführbarkeit werden alle in das Programm einzuschließende 1070 (1124 einschließlich Drop Outs) CAYAs in der Auswertungsphase analysiert (Gesamtkohorte). Die Auswertung des zweiten primären Endpunktes zu den modularen Interventionen erfolgt von allen vorliegenden 12 Monatsdaten der randomisierten CAYAs mit hohem Bedarf (266 angestrebt). In der Kohorte der CAYAs mit mindestens zwei verfügbaren konsekutiven Bedarfsanalysen, die die Kohorte der im Rahmen der initialen Studienphase randomisierten CAYAs einschließt, werden alle sekundären Endpunkte analysiert.

Im Rahmen der initialen Studienphase wird der primäre Endpunkt „Anteil Patienten mit hohem Interventionsbedarf“ mittels Likelihood-Ratio-Chi²-Test zwischen den Gruppen verglichen. Als Effektmaße werden absolute und relative Risikounterschiede mit 95% Konfidenzintervallen berichtet. Logistische Regressionsmodelle werden für adjustierte Analysen verwendet. In einem hierarchischen Test-Verfahren wird bei signifikantem Test des primären Endpunkts der Co-primäre Endpunkt „Rate der CAYAs mit noch nicht im Rahmen der Bedarfsanalyse charakterisierter Bedürfnisse“ ebenfalls mittels Likelihood-Ratio-Chi²-Test zwischen der Gruppe mit initialer Bedarfsanalyse (1.-535. CAYA) und der Gruppe der adaptierten Bedarfsanalyse (535.-1070. CAYA) verglichen. Als Effektmaße werden absolute und relative Risikounterschiede mit 95% Konfidenzintervallen berichtet.

Sekundäre dichotome Endpunkte werden analog analysiert, Für stetige Endpunkte werden parametrische oder, falls Annahmen der parametrischen Modelle nicht erfüllt sind, nicht-parametrische Testverfahren eingesetzt. P- Werte ≤ 0,05 werden als statistisch signifikant betrachtet. Alle Analysen werden mit SPSS (IBM Corp) oder R (R Foundation for Statistical Computing) in der zum Zeitpunkt der Auswertung aktuellen Version durchgeführt.

Neben dem Vergleich der Raten im Rahmen der initialen Studienphase sollen deskriptiv die Kosteneffizienz (gemessen an Personalkosten in Relation zur Effektivität des Interventionsprogramms) und hinsichtlich des Gesamtprogramms die Durchführbarkeit (Drop-out-Raten), die Veränderungen der Bedarfsrate bezogen auf das Gesamtprogramm und die einzelnen Module, sowie weitere modulspezifische Parameter (z.B. Verbesserung der Ernährung, gemessen am HEI-EPIC und Kurzscreening Ernährung vor und nach der Ernährungsintervention) untersucht werden. Darüber hinaus werden die in der Bedarfsanalyse eingesetzten, validierten Fragebögen entsprechend den publizierten Vorgaben ausgewertet und analysiert. Die Auswertung der Studienergebnisse wird in Hamburg am UKE durchgeführt.

Erhebung sekundärer Krankheitskosten zur Bestimmung der Kosteneffizienz

Um die langfristige Kosteneffizienz des CARE for CAYA-Programmes bestimmen zu können, sollen die in Anspruch genommenen Gesundheitskosten (ambulante und stationäre Diagnostik oder Behandlung, Rezepte) im Langzeitverlauf nach Tumorerkrankungen ermittelt werden. Aus diesem Grund werden im Rahmen des Programmes mit Unterstützung der Techniker Krankenkasse (TK) von TK-versicherten Teilnehmern diese Gesundheitskosten erhoben und mit „Nicht-Krebsbetroffenen" für bestimmte Kriterien (Alter, Geschlecht, etc.) „gematchten" Versicherten verglichen.

Die Erhebung wird wie folgt durchgeführt: Nach Gabe des zusätzlichen Einverständnisses von Versicherten der TK werden deren Identifikationsdaten (Name, Geburtsdatum, Versicherungsnummer, Datum des Studieneintritts bzw. Therapieabschlusses) einmalig an die TK weitergegeben. Mittels dieser Daten werden die Abrechnungsdaten von in Anspruch genommenen Gesundheitsleistungen der letzten 4 Jahre bei der Krankenkasse identifiziert und in pseudonymisierter Form für die projektbegleitende Auswertung gesammelt. Diese Daten werden dann mit einer „gematchten" anonymisierten Kohorte von Nicht-Betroffenen verglichen. Nach der pseudonymsierten Sammlung der Abrechnungsdaten werden sämtliche Verbindungen zu der Krankenkassenakte des jeweiligen Patienten gelöscht, so dass die Krankenkassenakte nach diesem Vorgang keinerlei Verweise, Daten oder Verbindungen zu der Studienteilnahme des Patienten (Zustand der Akte wie vor der Abfrage) mehr enthält.

Bewertung der einzelnen Module im Rahmen der Sekundären Endpunkte

Die einzelnen Module werden hinsichtlich der Effektivität der Intervention bewertet und mit den angefallenen Kosten korreliert, vergleichend in der Interventions- als auch Kontrollgruppe (jeweils die Gruppe mit hohem Bedarf im jeweiligen Modul. Die Effektivitätsbeurteilung der einzelnen Module erfolgt anhand der Endpunkte (siehe Punkt 5.2) die im Rahmen der kontinuierlichen 12 Monatserhebung erfasst werden. Es werden folgende Veränderungen angenommen, um die Interventionen als effektiv zu betrachten. Die Evaluation der Interventionsmodule erfolgt im Rahmen der initialen Studienphase, ggf. werden danach Anpassungen vorgenommen (siehe Punkt 7.2.5).

<u>Sport</u>

Primärer Endpunkt:

- Steigerung der körperlichen Aktivität gemessen anhand des erweiterten BSA-Fragebogens und/oder des Aktivitätsmessers

Sekundäre Endpunkte:

- Steigerung der regelmäßigen körperlichen Aktivität auf mindestens 3 Tage die Woche mit insgesamt >150 Minuten/Woche moderate körperliche Aktivität und/oder >75 Minuten intensive körperliche Aktivität

- Abbau der situativen und sportbezogenen Barrieren
- Verbesserung der Lebensqualität und Fatigue (EORTC QLQ-C30 und FA12)
- Verbesserung des Phasenwinkels um 5% im Vergleich zur Baseline-Messung
- Verbesserung der Ausdauerleistungsfähigkeit gemessen im Rahmen der Spiroergometrie und Laktatdiagnostik (Subgruppe an einzelnen Zentren)

Ernährung

Primärer Endpunkt:

- Verbesserung des Ernährungsverhaltens im Vergleich zur Baseline-Messung gemessen mittels HEI-EPIC und Kurzscreening Ernährung

Sekundäre Endpunkte:

- Verbesserung des Phasenwinkels um 5% im Vergleich zur Baseline-Messung
- Verbesserung des MEDAS-FFQ
- Verminderung der Anzahl an Patienten, die nach BMI Untergewicht, Übergewicht oder Adipositas haben im Vergleich zur Baseline-Messung
- Abbau der ernährungsbezogenen Barrieren
- Verbesserung der Lebensqualität und Fatigue (EORTC QLQ-C30 und FA12)
- Verbesserung von subjektiven und objektiven Geschmackwahrnehmung (Subgruppe an einzelnen Zentren)

Psychoonkologie

Primärer Endpunkt:

- Erhöhung der Patientenkompetenz (CBI-B)

Sekundäre Endpunkte:

- Erhöhung des Selbstwirksamkeitserlebens (SWE)
- Reduktion der Depressivität (PHQ-9)
- Reduktion des Distresserlebens (NCCN DT)
- Reduktion der Ängstlichkeit (GAD-7)
- Reduktion der Progredienzangst (PA-F-KF)
- Verbesserung der Lebensqualität und Fatigue (EORTC QLQ-C30 und FA12)

a. Fallzahlkalkulation

Primärer Endpunkt (Effektivität des Programms): Innerhalb der Gruppe von CAYAs mit hohem Bedarf wird bereits eine Verminderung des Interventionsbedarfs durch die Basisversorgung erwartet (maximal 10%). Die weiterführenden, bedarfsadaptierten Interventionen im Rahmen des *CARE for CAYA-Programms* sollen den Interventionsbedarf in dieser Gruppe von 90% um zusätzliche 15% auf damit 75% der CAYAs mit initial hohen Bedarf nach einem Zeitraum von 12 Monaten reduzieren. Bei einer 1:1 Randomisierung unter Anwendung des Likelihood-Ratio-Chi²-Tests und unter Berücksichtigung eines Alpha- von 5% und eines Beta-Fehlers von 10% müssen dafür von insgesamt 266 CAYAs die 12 Monatsevaluationen vorliegen. Bei einer Drop-out-Rate von ca. 20% werden insgesamt 320 CAYAs mit

initial hohem Bedarf randomisiert. Die Fallzahlberechnung erfolgte mittels NCSS PASS 2008. In Anbetracht der o.g. Verteilung von ca. 60% CAYAs mit hohem Bedarf müssen für den klinischen Studienteil ca. 530 CAYAs rekrutiert werden.

Co-primärer Endpunkt (Bedarfsanalyse)

Basierend auf eigenen Vordaten im Rahmen unserer kontinuierlichen fragebogenbasierten Bedarfsanalyse von Krebsüberlebenden zeigte sich, dass bei 20% der Befragten über die Bedarfsanalyse hinausgehende und nicht entsprechend charakterisierte Bedürfnisse bestehen. Demnach ist für die initiale Bedarfsanalyse bis zum 535. CAYA von dieser Rate auszugehen. Durch Adaptation der Bedarfsanalyse im Verlauf soll diese Rate bei der zweiten Gruppe (536. bis 1070. CAYA) um ungefähr ein Drittel auf 13,6% reduziert werden. Unter Anwendung eines zweiseitigen Likelihood-Ratio-Chi^2-Tests und unter Berücksichtigung eines alpha-Fehlers von 5% und eines Beta-Fehlers von 20% (80% Power) müssen dafür von insgesamt 2x535, also 1070 CAYAs die Bedarfsanalysen vorliegen. Unter Berücksichtigung einer 5% Drop out Rate ist der Einschluss von 1124 Krebsüberlebenden geplant.

Das **gesamte *CARE for CAYA-Programm*** *wird* für 3 Jahre rekrutieren (Zeitraum der Finanzierung durch den GBA Innovationsfonds). In diesem Zeitraum ist der Einschluss von 1124 CAYAs geplant.

8. Datenmanagement

Zu Beginn des *CARE for CAYA-Programm*s erhalten alle CAYAs einen Basisfragebogen (wie oben beschrieben) in elektronischer Form (auf einem Tablet) hinterlegt oder in Papierform. Die Tablets mit dem hinterlegten Fragebogen werden jedem Konsortialpartner zur Verfügung gestellt.

Jedem teilnehmendem CAYA wird eine Identifikationsnummer (Pat-ID) zugewiesen. Nach Beantwortung aller Fragen werden diese an den Zentralserver des Studienzentrums (UCCH) in pseudonymisierter Form weitergeleitet. Diese Daten werden in einer passwortgeschützten Studiendatenbank (Forschungsdatenbank des ICB-L der Universität zu Lübeck) gespeichert, zu der nur der Studienleiter PD. Dr. med. A. Stein und die von ihm mit der Auswertung und Dateneingabe beauftragten Personen des UCCHs oder der Konsortialzentren Zugang haben.

Voraussetzung für die Studienteilnahme und die Verwendung der Daten ist das Vorliegen einer schriftlichen Einverständniserklärung. Die erhobenen Daten werden für 15 Jahre archiviert und danach vernichtet. Sofern notwendig, können alle Daten und Studiendokumente in anonymisierter Form zur Qualitätskontrolle durch externe Gutachter überprüft werden.

Mit dem Einverständnis des Patienten werden die persönlichen pseudonymisierten Daten lediglich in anonymisierter Form weitergegeben oder veröffentlicht, so dass eine Zuordnung zur Person unmöglich wird.

Bei Widerruf der Einwilligung des Patienten zur Teilnahme an der klinischen Studie werden die schon gewonnenen pseudonymisierten Daten aus dem Register gelöscht. Falls die Daten in anonymisierter Form weitergegeben worden sind, können diese zwar nicht mehr nachträglich gelöscht werden, eine Zuordnung zu einer Person kann hierbei aber ausgeschlossen werden.

Die Studie ist in dem deutschen Register klinischer Studien (DRKS) eingetragen.

Ergebnisse der Studie werden in nationalen und internationalen Zeitschriften mit Peer-Review veröffentlicht. Vorgesehen sind außerdem Vorstellungen der Ergebnisse auf nationalen und internationalen Kongressen in Form von Vorträgen oder Postern.

9. Abkürzungsverzeichnis

ALL	Akute lymphatische Leukämie
AML	Akute Myeloische Leukämie
BIA	Bioimpedanzanalyse
BMI	Body Mass Index
CAYA	Children, Adolescents and Young Adults
COSS	Cooperative Osteosarkom Studiengruppe
DASH	Dietary Approaches to Stop Hypertension
DGE	Deutsche Gesellschaft für Ernährung
DGHO	Deutsche Gesellschaft für Hämatologie und Medizinische Onkologie
DKG	Deutsche Krebsgesellschaft
GBA	Gemeinsamer Bundesausschuss
GEKID	Gesellschaft der epidemiologischen Krebsregister in Deutschland
GPOH	Gesellschaft für Pädiatrische Onkologie und Hämatologie
INAYA	Improved Nutrition in AYAs
KHK	Koronare Herzerkrankungen
LESS	Late Effects Surveillance System
MAYA	Motivate AYA
MET	metabolisches Äquivalent
NCCN	National Comprehensive Cancer Network
NHL	Non-Hodgkin-Lymphom
TK	Techniker Krankenkasse
WCRF	World Cancer Research Fund
WHR	Waist-Hip-Ratio
WPO	Weiterbildung Psychosoziale Onkologie
ZfKD	Zentrum für Krebsregisterdaten

10. Literaturverzeichnis

1. *Krebs in Deutschland 2011/2012*. 2015, Robert Koch-Institut (Hrsg) und die Gesellschaft der epidemiologischen Krebsregister in Deutschland e.V. (Hrsg): Berlin.
2. Hilgendorf, I., et al. *Heranwachsende und junge Erwachsene (AYA, Adolescents and Young Adults)*. Januar 2016; Available from: www.onkopedia.com.
3. Kuten, J., et al., *Plasma Levels of Heparanase as a Marker of Tumor Progression and Aggressiveness in Patients with Colorectal and Breast Cancer.* Annals of Oncology, 2010. **21**: p. 76-76.
4. Pritzkuleit, R. *Auswertung der Krebshäufigkeit für die Leitlinie „Heranwachsende und junge Erwachsene (AYA, Adolescents and Young Adults)"*. Dezember 2014; Available from: https://www.onkopedia.com/de/wissensdatenbank/wissensdatenbank/heranwachsende-und-junge-erwachsene-aya-adolescents-and-young-adults/Pritzkuleit%202015.pdf.
5. Creutzig, U., et al., *Krebserkrankungen bei Kindern - Erfolg durch einheitliche Therapiekonzepte seit 25 Jahren.* Deutsches Ärzteblatt, 2003(13): p. 842-852.
6. Robison, L.L. and M.M. Hudson, *Survivors of childhood and adolescent cancer: life-long risks and responsibilities.* Nat Rev Cancer, 2014. **14**(1): p. 61-70.
7. Armstrong, G.T., et al., *Reduction in late mortality among 5-year survivors of childhood cancer: A report from the Childhood Cancer Survivor Study (CCSS).* Journal of Clinical Oncology, 2015. **33**(15).
8. Yang, L.M. and J. Fujimoto, *Childhood cancer mortality in Japan, 1980-2013.* Bmc Cancer, 2015. **15**.
9. Oeffinger, K.C., et al., *Chronic health conditions in adult survivors of childhood cancer.* New England Journal of Medicine, 2006. **355**(15): p. 1572-1582.
10. Mulrooney, D.A., et al., *Cardiac outcomes in a cohort of adult survivors of childhood and adolescent cancer: retrospective analysis of the Childhood Cancer Survivor Study cohort.* Bmj, 2009. **8**(339).
11. Tukenova, M., et al., *Role of cancer treatment in long-term overall and cardiovascular mortality after childhood cancer.* J Clin Oncol, 2010. **28**(8): p. 1308-15.
12. Armstrong, G.T., et al., *Modifiable risk factors and major cardiac events among adult survivors of childhood cancer.* J Clin Oncol, 2013. **31**(29): p. 3673-80.
13. Lipshultz, S.E., et al., *Long-term cardiovascular toxicity in children, adolescents, and young adults who receive cancer therapy: pathophysiology, course, monitoring, management, prevention, and research directions: a scientific statement from the American Heart Association.* Circulation, 2013. **128**(17): p. 1927-95.
14. Rath, H.M., et al., *Psychometric properties of the Occupational Stress and Coping Inventory (AVEM) in a cancer population.* Acta Oncol, 2015. **54**(2): p. 232-42.
15. Schrag, N.M., et al., *Stress-related mental disorders in childhood cancer survivors.* Pediatr Blood Cancer, 2008. **50**(1): p. 98-103.
16. Faller, H., et al., *Satisfaction with information and unmet information needs in men and women with cancer.* J Cancer Surviv, 2016. **10**(1): p. 62-70.
17. Cadman, D., et al., *Chronic illness, disability, and mental and social well-being: findings of the Ontario Child Health Study.* Pediatrics, 1987. **79**(5): p. 805-13.
18. Schmidt, J.E., et al., *Prevalence of perceived cognitive dysfunction in survivors of a wide range of cancers: results from the 2010 LIVESTRONG survey.* J Cancer Surviv, 2016. **10**(2): p. 302-11.
19. Kanellopoulos, A., et al., *Neurocognitive Outcome in Very Long-Term Survivors of Childhood Acute Lymphoblastic Leukemia After Treatment with Chemotherapy Only.* Pediatr Blood Cancer, 2016. **63**(1): p. 133-8.
20. Schmiegelow, K., et al., *Second malignant neoplasms after treatment of childhood acute lymphoblastic leukemia.* J Clin Oncol, 2013. **31**(19): p. 2469-76.
21. Mulrooney, D.A., et al., *Cardiac outcomes in a cohort of adult survivors of childhood and adolescent cancer: retrospective analysis of the Childhood Cancer Survivor Study cohort.* British Medical Journal, 2009. **339**.

22. Castellino, S.M., et al., *Morbidity and mortality in long-term survivors of Hodgkin lymphoma: a report from the Childhood Cancer Survivor Study.* Blood, 2011. **117**(6): p. 1806-1816.
23. Chao, C., et al., *Cardiovascular Disease Risk Profiles in Survivors of Adolescent and Young Adult (AYA) Cancer: The Kaiser Permanente AYA Cancer Survivors Study.* J Clin Oncol, 2016.
24. Chow, E.J., et al., *Individual prediction of heart failure among childhood cancer survivors.* J Clin Oncol, 2015. **33**(5): p. 394-402.
25. Rock, C.L., et al., *Nutrition and physical activity guidelines for cancer survivors.* CA Cancer J Clin, 2012. **62**(4): p. 243-74.
26. Eckel, R.H., et al., *2013 AHA/ACC guideline on lifestyle management to reduce cardiovascular risk: a report of the American College of Cardiology/American Heart Association Task Force on Practice Guidelines.* J Am Coll Cardiol, 2014. **63**(25 Pt B): p. 2960-84.
27. Srinath Reddy, K. and M.B. Katan, *Diet, nutrition and the prevention of hypertension and cardiovascular diseases.* Public Health Nutr, 2004. **7**(1A): p. 167-86.
28. Jones, L.W., et al., *Exercise and risk of major cardiovascular events in adult survivors of childhood hodgkin lymphoma: a report from the childhood cancer survivor study.* J Clin Oncol, 2014. **32**(32): p. 3643-50.
29. Guy, G.P., Jr., et al., *Estimating the health and economic burden of cancer among those diagnosed as adolescents and young adults.* Health Aff (Millwood), 2014. **33**(6): p. 1024-31.
30. Brown, M.L., et al., *Estimating health care costs related to cancer treatment from SEER-Medicare data.* Med Care, 2002. **40**(8 Suppl): p. IV-104-17.
31. Parsons, H.M., et al., *Impact of cancer on work and education among adolescent and young adult cancer survivors.* J Clin Oncol, 2012. **30**(19): p. 2393-400.
32. Finkelstein, E.A., et al., *The personal financial burden of cancer for the working-aged population.* Am J Manag Care, 2009. **15**(11): p. 801-6.
33. Moran, J.R., P.F. Short, and C.S. Hollenbeak, *Long-term employment effects of surviving cancer.* J Health Econ, 2011. **30**(3): p. 505-14.
34. Dowling, E.C., et al., *Lost productivity and burden of illness in cancer survivors with and without other chronic conditions.* Cancer, 2013. **119**(18): p. 3393-401.
35. Schmitz, K.H., et al., *American College of Sports Medicine roundtable on exercise guidelines for cancer survivors.* Med Sci Sports Exerc, 2010. **42**(7): p. 1409-26.
36. Brown, J.C., et al., *Efficacy of exercise interventions in modulating cancer-related fatigue among adult cancer survivors: a meta-analysis.* Cancer Epidemiol Biomarkers Prev, 2011. **20**(1): p. 123-33.
37. Speck, R.M., et al., *An update of controlled physical activity trials in cancer survivors: a systematic review and meta-analysis.* J Cancer Surviv, 2010. **4**(2): p. 87-100.
38. Lollgen, H. and D. Lollgen, *Risk reduction in cardiovascular diseases by physical activity.* Internist, 2012. **53**(1): p. 20-+.
39. Sofi, F., et al., *Physical activity during leisure time and primary prevention of coronary heart disease: an updated meta-analysis of cohort studies.* Eur J Cardiovasc Prev Rehabil, 2008. **15**(3): p. 247-57.
40. Reiner, M., et al., *Long-term health benefits of physical activity--a systematic review of longitudinal studies.* BMC Public Health, 2013. **13**: p. 813.
41. Jones, L.W., et al., *Exercise and Risk of Cardiovascular Events in Women With Nonmetastatic Breast Cancer.* J Clin Oncol, 2016.
42. Murnane, A., et al., *Adolescents and young adult cancer survivors: exercise habits, quality of life and physical activity preferences.* Support Care Cancer, 2015. **23**(2): p. 501-10.
43. Barnes, M., K. Casazza, and H. Austin, *Strategies to promote regular exercise in adolescent and young adult cancer survivors.* Clinical Oncology in Adolescents and Young Adults, 2015: p. 103.
44. Wolin, K.Y., et al., *Implementing the exercise guidelines for cancer survivors.* J Support Oncol, 2012. **10**(5): p. 171-7.
45. Mayer, D.K., et al., *Health Behaviors in Cancer Survivors.* Oncology Nursing Forum, 2007. **34**(3): p. 643-651.

46. Quidde, J., et al., *Improved Nutrition in Adolescents and Young Adults after childhood cancer: INAYA—A pilot study*, in *Cancer Survivorship Symposium: Advancing Care and Research*. 2016, J Clin Oncol.
47. Micha, R., et al., *Estimating the global and regional burden of suboptimal nutrition on chronic disease: methods and inputs to the analysis.* European Journal of Clinical Nutrition, 2012. **66**(1): p. 119-129.
48. Ros, E., et al., *Mediterranean diet and cardiovascular health: Teachings of the PREDIMED study.* Adv Nutr, 2014. **5**(3): p. 330S-6S.
49. Farvid, M.S., et al., *Dietary protein sources in early adulthood and breast cancer incidence: prospective cohort study.* Bmj, 2014. **348**: p. g3437.
50. Gonzalez, C.A. and E. Riboli, *Diet and cancer prevention: where we are, where we are going.* Nutr Cancer, 2006. **56**(2): p. 225-31.
51. Sinha, R., et al., *Meat intake and mortality: a prospective study of over half a million people.* Arch Intern Med, 2009. **169**(6): p. 562-71.
52. *Food, Nutrition, Physical Activity, and the Prevention of Cancer: a Global Perspective.* 2007, World Cancer Research Fund / American Institute for Cancer Research: Washington DC.
53. World Cancer Research Fund, A.I.f.C.R.H., *Ernährung, körperliche Aktivität und Krebsprävention: Eine globale Perspektive. Zusammenfassung. World Cancer Research Fund International.* 2007.
54. Cook, S.L., et al., *Effect of nutrition counselling on client perceptions and eating behaviour.* Can J Diet Pract Res, 2006. **67**(4): p. 171-7.
55. Ammerman, A., et al., in *Counseling to Promote a Healthy Diet*. 2002: Rockville (MD).
56. Keyserling, T.C., et al., *A comparison of live counseling with a web-based lifestyle and medication intervention to reduce coronary heart disease risk: a randomized clinical trial.* JAMA Intern Med, 2014. **174**(7): p. 1144-57.
57. Bader N, B.M., Pfeiffer K, Schneider J, Meister A, Voigt S, Kohlenberg-Müller K, *Leben mit Brustkrebs – Ernährung in der rezidiv-freien stabilen Lebensphase.* Ernährungs Umschau, 2014. **61(3)**: p. 42-49.
58. Cohen, J., et al., *Taste and smell dysfunction in childhood cancer survivors.* Appetite, 2014. **75**: p. 135-140.
59. Cadman D, et al., *Chronic illness, disability, and mental and social well-being: findings of the Ontario Child Health Study.* Pediatrics, 1987. **79**(5): p. 805-13.
60. Langer, T., et al., *Die Überlebenden einer Krebserkrankung im Kindesalter -Nachsorge und Spätfolgen nach erfolgreicher Therapie.* Monatsschrift Kinderheilkunde, 2002. **150**: p. 942-953.
61. Seitz, D., et al., *Life satisfaction in adult survivors of cancer during adolescence: what contributes to the latter satisfaction with life?* Quality of Life Research, 2011. **20**(2): p. 225-236.
62. Engelen, V., et al., *Health-related quality of life after completion of successful treatment for childhood cancer.* Pediatric Blood & Cancer, 2011. **56**(4): p. 646-653.
63. Reinfjell, T., et al., *Children in remission from acute lymphoblastic leukaemia: mental health, psychosocial adjustment and parental functioning.* European Journal of Cancer Care, 2009. **18**(4): p. 364-370.
64. Barrera, M., et al., *Educational and social late effects of childhood cancer and related clinical, personal, and familial characteristics.* Cancer, 2005. **104**(8): p. 1751-1760.
65. Bottcher, H.M., et al., *Work-related predictors of not returning to work after inpatient rehabilitation in cancer patients.* Acta Oncol, 2013. **52**(6): p. 1067-75.
66. Langer, T., et al., *Die Überlebenden einer Krebserkrankung im Kindesalter - Nachsorge und Spätfolgen nach erfolgreicher Therapie.* Monatsschrift Kinderheilkunde, 2002. **150**: p. 942-953.
67. Wenninger, K., et al., *Coping in long-term survivors of childhood cancer: relations to psychological distress.* Psycho-Oncology, 2013. **22**(4): p. 854-861.
68. Schrag, N.M., et al., *Stress-related mental disorders in childhood cancer survivors.* Pediatric Blood & Cancer, 2008. **50**(1): p. 98-103.

69. Faller, H., et al., *Satisfaction with information and unmet information needs in men and women with cancer.* J Cancer Surviv, 2015.
70. Sarkar, S., et al., *Anxiety and fear of cancer recurrence and its association with supportive care needs and health-care service utilization in cancer patients.* J Cancer Surviv, 2015.
71. Akechi, T., et al., *Patient's perceived need and psychological distress and/or quality of life in ambulatory breast cancer patients in Japan.* Psychooncology, 2011. **20**(5): p. 497-505.
72. Miller W.R., R.S., *Motivational Interviewing. Preparing people for change. Second edition.* 2002, New York: Guilford.
73. Körkel J, V.C., *Motivational Interviewing: Eine Übersicht.* Suchttherapie. Vol. 4. 2003.
74. Spencer, J.C. and S.B. Wheeler, *A systematic review of Motivational Interviewing interventions in cancer patients and survivors.* Patient Educ Couns, 2016. **99**(7): p. 1099-105.
75. Cella, D., et al., *Cancer-related fatigue: prevalence of proposed diagnostic criteria in a United States sample of cancer survivors.* J Clin Oncol, 2001. **19**(14): p. 3385-91.
76. Edwards, J.L., et al., *Fatigue in adolescents with and following a cancer diagnosis: developing an evidence base for practice.* Eur J Cancer, 2003. **39**(18): p. 2671-80.
77. Campos, M.P., et al., *Cancer-related fatigue: a practical review.* Ann Oncol, 2011. **22**(6): p. 1273-9.
78. Langeveld, N., et al., *'I don't have any energy': The experience of fatigue in young adult survivors of childhood cancer.* Eur J Oncol Nurs, 2000. **4**(1): p. 20-8.
79. Hooke, M.C., A.W. Garwick, and C.R. Gross, *Fatigue and physical performance in children and adolescents receiving chemotherapy.* Oncol Nurs Forum, 2011. **38**(6): p. 649-57.
80. Perdikaris, P., et al., *Evaluating cancer related fatigue during treatment according to children's, adolescents' and parents' perspectives in a sample of Greek young patients.* Eur J Oncol Nurs, 2009. **13**(5): p. 399-408.
81. Sarkar, S., et al., *Anxiety and fear of cancer recurrence and its association with supportive care needs and health-care service utilization in cancer patients.* J Cancer Surviv, 2015. **9**(4): p. 567-75.
82. Quidde, J., et al., *Cancer Survivorship – First Experiences of the L.O.T.S.E. – Project of the University Cancer Center Hamburg (UCCH)*, in *European Cancer Rehabilitation and Survivorship Symposium, Copenhagen*. 2014. p. 66-67.
83. Creutzig, U., et al., *[Concepts of the Society of Paediatric Oncology and Haematology (GPOH) and the German Competence Network in Paediatric Oncology and Haematology for the quality controlled development in paediatric oncology].* Klin Padiatr, 2004. **216**(6): p. 379-83.
84. Schröder, H.M., et al., *Psychosoziale Versorgung in der Pädiatrischen Onkologie und Hämatologie (AWMF-Leitlinie)*. 2013, Psychosoziale Arbeitsgemeinschaft in der Pädiatrischen Onkologie und Hämatologie (PSAPOH).
85. Berdan, C.A., et al., *Childhood cancer survivors and adherence to the American Cancer Society Guidelines on Nutrition and Physical Activity.* J Cancer Surviv, 2014. **8**(4): p. 671-9.
86. M., K., *Adoleszente und junge Erwachsene mit Krebs - Psychoonkologische Aspekte der medizinischen Versorgung. .* Onkologe, 2015(21): p. 953-958.
87. Gupta, A.A., et al., *Reimagining care for adolescent and young adult cancer programs: Moving with the times.* Cancer, 2016. **122**(7): p. 1038-46.
88. Pugh, G., et al., *Health Behavior Change Interventions for Teenage and Young Adult Cancer Survivors: A Systematic Review.* J Adolesc Young Adult Oncol, 2016.
89. Thompson, K., S. Palmer, and G. Dyson, *Adolescents & young adults: Issues in transition from active therapy into follow-up care.* European Journal of Oncology Nursing, 2009. **13**(3): p. 207-212.
90. Grundherr, J.v., et al., *Effektivität einer Intervention zur Verbesserung des Ernährungsverhaltens von jungen Patienten nach überstandener Krebserkrankung im Kindes-, Jugend- und jungen Erwachsenenalter (AYAs) mit kardiovaskulärem Risiko – INAYA Studie.* Aktuel Ernahrungsmed, 2016. **41**(03): p. P22.
91. Fuchs, R., et al., *Messung der Bewegungs- und Sportaktivität mit dem BSA-Fragebogen.* Zeitschrift für Gesundheitspsychologie, 2015. **23**(2): p. 60-76.

92. Knobel, H., et al., *The validity of EORTC QLQ-C30 fatigue scale in advanced cancer patients and cancer survivors.* Palliative Medicine, 2003. **17**(8): p. 664-672.
93. Heitzmann, C.A., et al., *Assessing self-efficacy for coping with cancer: development and psychometric analysis of the brief version of the Cancer Behavior Inventory (CBI-B).* Psycho-Oncology, 2011. **20**(3): p. 302-312.
94. Weinman, J., Wright S., Johnston, M., *Measures in health psychology: A user's portfolio. Causal and control beliefs.* Generalized Self-Efficacy scale, ed. R. Schwarzer, Jerusalem, M. . 1995, Windsor NFER-NELSON.
95. Kroenke, K., R.L. Spitzer, and J.B. Williams, *The PHQ-9: validity of a brief depression severity measure.* J Gen Intern Med, 2001. **16**(9): p. 606-13.
96. Anja Mehnert, D.M., Claudia Lehmann und Uwe Koch, *Die deutsche VersiondesNCCN Distress-Thermometers.* Zeitschrift für Psychiatrie, Psychologie und Psychotherapie, 2006. **54 (3)**: p. 213–223.
97. Spitzer, R.L., et al., *A brief measure for assessing generalized anxiety disorder: the GAD-7.* Arch Intern Med, 2006. **166**(10): p. 1092-7.
98. Mehnert, A., et al., *Fear of progression in breast cancer patients - validation of the short form of the Fear of Progression Questionnaire (FoP-Q-SF).* Zeitschrift Fur Psychosomatische Medizin Und Psychotherapie, 2006. **52**(3): p. 274-288.
99. Aaronson, N.K., et al., *The European Organization for Research and Treatment of Cancer QLQ-C30: a quality-of-life instrument for use in international clinical trials in oncology.* J Natl Cancer Inst, 1993. **85**(5): p. 365-76.
100. Akbar, A., et al., *Development of Prediction Model and Experimental Validation in Predicting the Curcumin Content of Turmeric (Curcuma longa L.).* Front Plant Sci, 2016. **7**: p. 1507.
101. Löllgen, *Das Anstrengungsempfinden (RPE, Borg-Skala).* DEUTSCHE ZEITSCHRIFT FÜR SPORTMEDIZIN, 2004. **Jahrgang 55**(Nr. 11).
102. Lowe, B., et al., *A 4-item measure of depression and anxiety: validation and standardization of the Patient Health Questionnaire-4 (PHQ-4) in the general population.* J Affect Disord, 2010. **122**(1-2): p. 86-95.
103. Schmidt, J., F. Lamprecht, and W.W. Wittmann, *Satisfaction with Inpatient Care Development of a Questionnaire and 1st Validity Assessments.* Psychotherapie Psychosomatik Medizinische Psychologie, 1989. **39**(7): p. 248-255.
104. Herdmann, M., *Development and preliminary testing of the new five-level version of EQ-5D (EQ-5D-5L).* Qual Life Res. , 2011. **Dec;20(10):1727-36. doi: 10.1007/s11136-011-9903-x.**
105. Egger, N., et al., *Short-term cost-effectiveness of psychodynamic therapy and cognitive-behavioral therapy in social anxiety disorder: Results from the SOPHO-NET trial.* Journal of Affective Disorders, 2015. **180**: p. 21-28.
106. Chisholm, D., et al., *Client Socio-Demographic and Service Receipt Inventory--European Version: development of an instrument for international research. EPSILON Study 5. European Psychiatric Services: Inputs Linked to Outcome Domains and Needs.* Br J Psychiatry Suppl, 2000(39): p. s28-33.
107. Eigenentwicklung universitäres cancer center, U., *Inanspruchnahme medizinischer Leistungen, Auszug* Stand 03.11.2017.
108. Eigenentwicklung Institut für medizinische Psychologie, U., *Inanspruchnahme psychoonkologischer Leistungen, Auszug.* Stand 03.11.2017.
109. Girgis, A., et al., *The next generation of the supportive care needs survey: a brief screening tool for administration in the clinical oncology setting.* Psychooncology, 2012. **21**(8): p. 827-35.
110. Basler, H.-D., *Selbstwirksamkeit, Entscheidungsbalance und die Motivation zu sportlicher Aktivität* Zeitschrift für Differentielle und Diagnostische Psychologie 1999. **20**: p. pp. 203-216.
111. Krämer, L.u.F., R., *Skalen zu den sportbezogenen situativen Barrieren und dem sportbezogenen Barrieremanagement.* 2009, Albert-Ludwigs-Universität: Freiburg.
112. Keller, *Zur Validität des Transtheoretischen Modells: Eine Untersuchung zur Veränderung des Ernährungsverhaltens. Dissertation. Universität Marburg* 1998.

113. Hebestreit, K., et al., *Validation of the German version of the Mediterranean Diet Adherence Screener (MEDAS) questionnaire.* BMC Cancer, 2017. **17**(1): p. 341.

114. Fuchs, R.G., W.; Seelig, H.; Fleitz, A.; Mahler, C.; Schittich, I., *Lebensstil-integrierte sportliche Aktivität: Ergebnisse der MoVo-LISA Interventionsstudie.* Bewegungstherapie und Gesundheitssport, 2010(26: 270–276).

11. Anhang

12. CARE for CAYA-Einwilligungserklärung 15-17 Jahre und 18-39 Jahre
13. CARE for CAYA-Patienteninformation 15-17 Jahre und 18-39 Jahre
14. Basis- und Screeningfragebögen bestehend aus den Einzelbögen: NCCN Distress Thermometer (DT), Lebensqualität (EORTC QLQ C30), Freiburger Ernährungsprotokoll, Abfrage zu körperlicher Aktivität mit Borg-Skala (Screeningfragen Modul Sport), BSA-Fragebogen erweitert mit Borg-Skala, Ultrakurzform des Gesundheitsfragebogens für Patienten (PHQ-4), Abfrage von nicht adressiertem Unterstützungsbedarf, Abfrage des Bedarfs, EQ5D-5L , Auszug aus Fragebogen zu Arbeitsausfall bei Erwachsenen, Auszug aus Fragebogen zu Leistungsvermögen in Schule, Ausbildung und bei der Arbeit von Jugendlichen und jungen Erwachsenen, Kurzfragebogen Inanspruchnahme medizinischer Leistungen, SCNS-TF-9, Zufriedenheitsfragebogen ZUF-8, modifiziert
15. Erweiterte modulspezifische Fragebögen bestehend aus: Angstmodul des Gesundheitsfragebogens für Patienten (GAD-7), Depressionsmodul des Gesundheitsfragebogens für Patienten (PHQ-9), Fatigue-Modul des EORTC (EORTC QLQ-FA12), Allgemeine Selbstwirksamkeitserwartung (SWE), Cancer Behavior Inventory – Brief Version (CBI-B), Progredienzangstfragebogen – Kurzversion (PA-F-KF), Fragen zur Einstellung zu körperlicher Aktivität (TTM), Skalen Sportbezogene situative Barrieren und Sportbezogenes Barrieremanagement, Bewegungstagebuch mit Borg-Skala, Fragen zur Einstellung zu Ernährungsumstellung (TTM), Entscheidungsbalance Kurzform, Fragebogen zu mediterranen Diät MEDAS-FFQ, Anamnesebogen zur Erhebung des Geruchs- und Geschmacksempfindens
