# Supplementary material for: A Multimodal Lifestyle Psychosocial Survivorship Program in Young Cancer Survivors: The CARE for CAYA Program—A Randomized Clinical Trial Embedded in a Longitudinal Cohort Study
Source: JAMA Netw Open. 2024 Mar 25;7(3):e242375. doi: 10.1001/jamanetworkopen.2024.2375 (PMC10964114; doi:10.1001/jamanetworkopen.2024.2375)
Supplement: Supplement 2. — eTable 1. Primary Outcome and Secondary Outcomes eTable 2. Module-Specific Interventions eTable 3. Module-Specific Primary Outcomes and High Need Criteria eTable 4. Adherence to Consultations eTable 5. Quality of Life and Fatigue (EORTC QLQ-C30) eTable 6. Prevalence and Development of Cardiovascular Risk Factors eTable 7. Persisting Module-Specific High Need [file jamanetwopen-e242375-s002.pdf]

Supplementary Online Content

von Grundherr J, Elmers S, Koch B, et al. A multimodal lifestyle psychosocial survivorship program in young cancer survivors: The CARE for CAYA Program–a randomized clinical trial embedded in a longitudinal cohort study. *JAMA Netw Open*. 2024;7(3):e242375. doi:10.1001/jamanetworkopen.2024.2375

eTable 1. Primary Outcome and Secondary Outcomes

eTable 2. Module-Specific Interventions

eTable 3. Module-Specific Primary Outcomes and High Need Criteria

eTable 4. Adherence to Consultations

eTable 5. Quality of Life and Fatigue (EORTC QLQ-C30)

eTable 6. Prevalence and Development of Cardiovascular Risk Factors

eTable 7. Persisting Module-Specific High Need

This supplementary material has been provided by the authors to give readers additional information about their work.

© 2024 von Grundherr J et al. *JAMA Network Open.*

eTable 1: Primary Outcome and Secondary Outcomes

Outcomes	Outcome description	Data Assessment
Primary outcome	Modular interventions *Rate of CAYAs (in %) with* high need for at least one modular intervention *after 12 months* (52 weeks) *of intervention (at least one criteria).*	**High need in Physical activity (PA) defined as:**
		< 150 min/week moderate physical activity and/or < 75 min intensive physical activity
		< 3 days physical activity per week
		Criteria of metabolic syndrome[a]
		< 2 days vigorous physical activity per week[b]
		**High need in Nutrition (NU) defined as:**
		≤ 40 HEI-EPIC-Score[c]
		Body Mass Index (BMI) < 18.5
		Gastrointestinal symptoms (e.g. diarrhea, nausea and vomiting, pain)
		Criteria of metabolic syndrome[a]
		≤ 29 points new Screening Tool: self-developed Short Healthy Eating Index Hamburg-2019 (SHEIH-19/21)[b]
		**High need in Psychooncology (PO) defined as:**
		≥ 6 points PHQ-4
		≥ 5 points NCCN-DT
Coprimary outcome	Need-stratified assessment *Rate of CAYAs with unmet needs that are outside of the scope of the assessment (comparison of initial assessment and adapted assessment)*	Quantitative survey: "In your opinion, are there any issues important to you that were not addressed in this list of questions?" [yes/no]
Secondary outcomes	Feasibility *Rate of establishment of the program in the consortium (existence of spatial conditions, staffing of personnel positions, implementation of the program process)*	Monthly inclusion rates of the centers
		Rate of CAYAs remaining in the program (needs assessment at least twice) in relation to participation in interventional modules
		Feasibility of implementing the adaptation of the needs assessment *(timeframe between evaluating the data, modifying the protocol, obtaining ethics committee approval, and implementing the adapted needs assessment)*
		Drop-out rates *(related to the overall study and the individual interventions)*
	Allocation and effectiveness of modular interventions	Evaluation of the need for intervention *Changes in needs after 52 weeks in relation to the need for intervention of the initial need and participation in an interventional module*
		Prevalence of cardiovascular risk factors (BMI, WHR, blood glucose/ lipid disorders, type of hypertension) *Evaluation of medical history data*
		Quality of life and fatigue *European Organization for Research and Treatment of Cancer QLQ-C30 (EORTC QLQ-C30)*
		Satisfaction *Modified questionnaire: satisfaction (ZUF-8)*

Abbreviations and explanations: PA = Physical Activity, NU= Nutrition, PO = Psychooncology…; [a] = Metabolic syndrome (at least one criteria): Diabetic metabolic disorder: Diabetes mellitus requiring insulin, Antidiabetics, HbA1c ≥ 6.5%, Fasting blood glucose ≥ 126 mg/dL. Lipid metabolism disorder: Lipid-lowering agents/Statins, Documented problems with lipid metabolism, Cholesterol total > 200 mg/dL, Cholesterol LDL > 150 mg/dL, Cholesterol HDL < 46 mg/dL, Triglycerides > 180 mg/dL. Arterial hypertension, Blood pressure drug, Documented arterial hypertension, Systolic blood pressure > 140 mmHg measured at least twice. BMI ≥ 30. Waist-Hip Ratio ≥ 0.85 (women), Waist-Hip Ratio ≥ 1.0 (men); [b] = Adapted inclusion criteria, added after RCT-Phase; [c] = The HEI-EPIC based on a 3-day dietary records and the score allows a classification in a good (≥ 65 points), moderate (>40-64 points), or poor (≤40 points) dietary behavior.

© 2024 von Grundherr J et al. *JAMA Network Open.*

eTable 2: Module-Specific Interventions

Module	Intervention	Aim	Assessment during intervention
Physical Activity	5x individual consultations about physical activity (week 0-24)	Improvement of physical activity behavior with the focus to strengthen the participants' ability to turn mere intentions into actual actions by individualized methods, for example collaborative goal setting	2x bio impedance analysis (BIA)
	9x newsletters about healthy diet and physical activity, (5x including individual recommendations)		Wearable activity monitoring over one week (ActiGraph)
Nutrition	5x individual consultations about healthy diet (week 0-24) based on the standardized German-Nutrition Care Process (G-NCP) including assessment, diagnosis, intervention, monitoring and evaluation	Individual advice for a healthy diet, how to maintain it in everyday life and how to overcome possible barriers were given	2x bio impedance analysis (BIA)
	1x shopping training (of 45-60 minutes), maximum 5 participants		
	1x cooking class (of 2-3 hours), maximum 8 participants		2x taste test (Taste Strips)
	9x newsletters about healthy diet and physical activity, (5x including individual recommendations)		
Psychooncology	5x motivational interviewing one-on-one sessions (week 0-16)	Reach their physical and mental goals by changing physical and mental coping behaviors	N/A
	Counselling sessions were carried out by a psychooncologist, who was trained in MI by the consortium leadership		
	5x newsletters about coping, self-care and mental health		

Abbreviation: N/A = Not applicable

eTable 3: Module-Specific Primary Outcomes and High Need Criteria

	Longitudinal study cohort	
	T1	T3
**Primary Outcome in Module: Physical Activity** Median (IQR)		
Moderate activity: Days per week	3.0 (2.0-5.0)	3.0 (2.0-5.0)
Moderate activity: Duration in minutes	45.0 (30.0-60.0)	45.0 (30.0-60.0)
Vigorous activity: Days per week	1.0 (0.0-3.0)	1.0 (0.0-2.0)
Vigorous activity: Duration in minutes	60.0 (30.0-75.0)	45.0 (30.0-60.0)
**Primary Outcome in Module: Nutrition**		
HEI-EPIC-Score [0-120]	48.0 (41.0-58.0)	52.0 (43.0-61.0)
**Primary Outcome in Module: Psychooncology**		
NCCN score [0-10]	5.0 (3.0-7.0)	5.0 (3.0-7.0)
PHQ-4 score [0-12]	2.0 (1.0-5.0)	2.0 (0.0-3.0)
**High need defining criteria in Module: Physical Activity** (No. (%))		
Questionnaire (PRO)	272/440 (61.8%)	91/264 (34.5%)
Metabolic syndrome: BMI	80/445 (18.0%)	44/233 (18.9%)
Metabolic syndrome: WHR	89/356 (25.0%)	14/148 (27.0%)
Metabolic syndrome: Labs	142/371 (31.6%)	68/158 (43.0%)
**High need defining criteria in Module: Nutrition**		
≤ 40 HEI-EPIC-Score (PRO)	111/391 (28.4%)	30/232 (12.9%)
BMI < 18.5	49/456 (10.7%)	20/243 (8.2%)
Gastrointestinal symptoms (PRO)	126/456[a] (27.6%)[a]	27/456[a] (5.9%)[a]
Metabolic syndrome: BMI	80/456 (17.5%)	44/243 (18.1%)
Metabolic syndrome: WHR	89/378 (23.5%)	40/147 (27.2%)
Metabolic syndrome: Labs	142/393 (36.1%)	68/156 (43.6%)
**High need defining criteria in Module: Psychooncology**		
≥ 6 points PHQ-4 (PRO)	134/457 (29.3%)	46/274 (16.8%)
≥ 5 points NCCN-DT (PRO)	445/460 (96.7%)	178/274 (65.0%)

Abbreviations and Explanations: PRO = Patient Reported Outcomes, IQR = Interquartile Range, Tx = Time of Measurement; [a] = Only value available.

© 2024 von Grundherr J et al. *JAMA Network Open.*

eTable 4: Adherence to Consultations

	Intervention Group						Control Group	
Consultations	0	1	2	3	4	5	0	1
**RCT Cohort** (No. (%))								
PA (101)	16 (15.8%)	6 (5.9%)	8 (7.9%)	5 (5.0%)	3 (3.0%)	63 (62.4%)	39 (31.2%)	86 (68.8%)
NU (109)	24 (22.0%)	7 (6.4%)	3 (2.8%)	4 (3.7%)	6 (5.5%)	65 (59.6%)	37 (29.4%)	89 (70.6%)
PO (121)	12 (9.9%)	10 (8.3%)	5 (4.1%)	6 (5.0%)	8 (6.6%)	80 (66.1%)	35 (28.2%)	89 (71.8%)
**Longitudinal study cohort** (No. (%))								
PA (145)	32 (22.1%)	11 (7.6%)	2 (1.4%)	4 (2.8%)	20 (13.8%)	76 (52.4%)	N/A[a]	N/A[a]
NU (160)	40 (25.0%)	7 (4.4%)	7 (4.4%)	5 (3.1%)	12 (7.5%)	89 (55.6%)	N/A[a]	N/A[a]
PO (161)	21 (13.0%)	12 (7.5%)	14 (8.7%)	6 (3.7%)	15 (9.3%)	93 (57.8%)	N/A[a]	N/A[a]

Abbreviations and Explanations: PA = Physical Activity, NU= Nutrition, PO = Psychooncology; [a] = Not applicable, because no Control Group after RCT-phase

© 2024 von Grundherr J et al. *JAMA Network Open.*

eTable 5: Quality of Life and Fatigue (EORTC QLQ-C30)

	Intervention Group		Control Group		Longitudinal study cohort	
	T1 (183)	T3 (138)	T1 (176)	T3 (136)	T1 (No.)	T3 (No.)
**EORTC QLQ-C30: total score (QoL)** median (IQR)	74.8 (63.3-84.0)	81.8 (67.8-92.3)	78.8 (63.9-87.8)	85.3 (74.1-94.3)	80.1 (66.4-90.2) (758)	86.5 (73.9-95.1) (455)
**EORTC QLQ-C30: Fatigue** median (IQR)	55.6 (33.3-66.7)	44.4 (22.2-66.7)	44.4 (33.3-66.7)	33.3 (22.2-55.6)	44.4 (22.2-66.7) (768)	33.3 (11.1-55.6) (460)

Abbreviation: Tx = Time of Measurement.

© 2024 von Grundherr J et al. *JAMA Network Open.*

eTable 6: Prevalence and Development of Cardiovascular Risk Factors

	Intervention Group		Control Group		*P*-value[a]
	T1 (183)	T3 (138)	T1 (176)	T3 (136)	
**Cardiovascular risk factors % (No. (%))**					
BMI ≥ 30	27/182 (14.8%)	19/117 (16.2%)	21/174 (12.1%)	16/113 (14.2%)	.66
WHR ≥ 0.85 (women), ≥ 1.0 (men)	24/149 (16.1%)	16/77 (20.8%)	28/145 (19.3%)	16/76 (21.1%)	.97
**Blood sugar/ lipid metabolism disorders % (No. (%))**					
Cholesterol total > 200 mg/dL	23/107 (21.5%)	7/48 (14.6%)	24/103 (23.3%)	12/49 (24.5%)	.22
Cholesterol LDL > 150 mg/dL	8/101 (7.9%)	3/49 (6.1%)	9/101 (8.9%)	4/49 (8.2%)	.69
Cholesterol HDL < 46 mg/dL	28/103 (27.2%)	20/49 (40.8%)	24/100 (24.0%)	15/49 (30.6%)	.29
Triglycerides > 180 mg/dL	19/102 (18.6%)	5/48 (10.4%)	14/98 (14.3%)	7/48 (14.6%)	.54
Lipid-lowering agents/Statins	3 (1.6%)	2/133 (1.5%)	0/175 (0.0%)	0/132 (0.0%)	N/A
Documented problems with lipid metabolism	9 (4.9%)	6/133 (4.5%)	5/175 (2.9%)	3/132 (2.3%)	.31
Lipid metabolism disorder	51/101 (50.5%)	30/52 (57.7%)	48/98 (49.0%)	24/49 (49.0%)	.38
Hyperlipidemia	6 (3.3%)	N/A	5/175 (2.9%)	N/A	N/A
Hypertriglyceridemia	5 (2.7%)	N/A	0/175 (0.0%)	N/A	N/A
Diabetic metabolic disorder	4/50 (8.0%)	2/16 (12.5%)	3/60 (5.0%)	2/22 (9.1%)	.74
Fasting blood glucose ≥ 126 mg/dL	1/56 (1.8%)	0/18 (0.0%)	0/66 (0.0%)	0/22 (0.0%)	N/A
HbA1c ≥ 6.5%	1/85 (1.2%)	1/41 (2.4%)	1/92 (1.1%)	0/47 (0.0%)	N/A
Arterial hypertension	14/137 (3.8%)	14/79 (17.7%)	16/38 (11.6%)	10/68 (14.7%)	.62
Systolic blood pressure > 140 mmHg	4/135 (3.0%)	5/76 (6.6%)	10/137 (7.3%)	2/66 (3.0%)	.32
Heart failure	6 (3.3%)	N/A	0/175 (0.0%)	N/A	N/A

Abbreviations and Explanations: Tx = Time of Measurement; [a] = difference between Intervention Group and Control Group; N/A = Not applicable

eTable 7: Persisting Module-Specific High Need

**Module** (No. (%))	**Intervention Group** (138)	**Control Group** (136)	**Odds Ratio (95% Confidence Interval)**	***P*-value**
Physical Activity	63/81 (77.0%)	62/89 (69.7%)	1.52 (0.77-3.08)	.23
Nutrition	74/103 (71.8%)	70/104 (67.3%)	1.24 (0.69-2.25)	.48
Psychooncology	78/111 (70.3%)	55/95 (57.9%)	1.72 (0.97-3.07)	.06

© 2024 von Grundherr J et al. *JAMA Network Open.*
