# Supplementary material for: A Multimodal Lifestyle Psychosocial Survivorship Program in Young Cancer Survivors: The CARE for CAYA Program—A Randomized Clinical Trial Embedded in a Longitudinal Cohort Study
Source: JAMA Netw Open. 2024 Mar 25;7(3):e242375. doi: 10.1001/jamanetworkopen.2024.2375 (PMC10964114; doi:10.1001/jamanetworkopen.2024.2375)
Supplement: Supplement 3. — Data Sharing Statement [file jamanetwopen-e242375-s003.pdf]

Data Sharing Statement

von Grundherr. A Multimodal Lifestyle Psychosocial Survivorship Program in Young Cancer Survivors. *JAMA Netw Open*. Published March 25, 2024.
doi:10.1001/jamanetworkopen.2024.2375

Data
Data available: No
